# Novel multitarget analgesic candidate SZV-1287 demonstrates potential disease-modifying effects in the monoiodoacetate-induced osteoarthritis mouse model

**DOI:** 10.3389/fphar.2024.1377081

**Published:** 2024-09-16

**Authors:** Ádám István Horváth, Kata Bölcskei, Nikolett Szentes, Éva Borbély, Valéria Tékus, Bálint Botz, Kitti Rusznák, Anett Futácsi, Boldizsár Czéh, Péter Mátyus, Zsuzsanna Helyes

**Affiliations:** ^1^ Department of Pharmacology and Pharmacotherapy, Medical School, University of Pécs, Pécs, Hungary; ^2^ National Laboratory for Drug Research and Development, Budapest, Hungary; ^3^ Hungarian Research Network, HUN-REN-PTE Chronic Pain Research Group, Pécs, Hungary; ^4^ Department of Laboratory Diagnostics, Faculty of Health Sciences, University of Pécs, Pécs, Hungary; ^5^ Department of Medical Imaging, Medical School, University of Pécs, Pécs, Hungary; ^6^ Department of Laboratory Medicine, Medical School, University of Pécs, Pécs, Hungary; ^7^ Neurobiology of Stress Research Group, János Szentágothai Research Centre, University of Pécs, Pécs, Hungary; ^8^ National Laboratory of Infectious Animal Diseases, Antimicrobial Resistance, Veterinary Public Health and Food Chain Safety, University of Veterinary Medicine, Budapest, Hungary; ^9^ PharmInVivo Ltd., Pécs, Hungary; ^10^ ALGONIST Biotechnologies GmBH, Vienna, Austria

**Keywords:** osteoarthritic pain, analgesic drug development, semicarbazide-sensitive amine oxidase, amine oxidase copper containing 3, vascular adhesion protein 1, transient receptor potential vanilloid 1, transient receptor potential ankyrin 1

## Abstract

**Introduction:**

Monoiodoacetate (MIA)-induced osteoarthritis (OA) is the most commonly used rodent model for testing anti-OA drug candidates. Herein, we investigated the effects of our patented multitarget drug candidate SZV-1287 (3-(4,5-diphenyl-1,3-oxazol-2-yl) propanal oxime) that is currently under clinical development for neuropathic pain and characterized the mouse model through complex functional, *in vivo* imaging, and morphological techniques.

**Methods:**

Knee OA was induced by intraarticular MIA injection (0.5 and 0.8 mg). Spontaneous pain was assessed based on weight distribution, referred pain by paw mechanonociception (esthesiometry), edema by caliper, neutrophil myeloperoxidase activity by luminescence, matrix metalloproteinase activity, vascular leakage and bone remodeling by fluorescence imaging, bone morphology by micro-CT, histopathological alterations by semiquantitative scoring, and glia activation by immunohistochemistry. Then, SZV-1287 (20 mg/kg/day) or its vehicle was injected intraperitoneally over a 21-day period.

**Results:**

MIA induced remarkable weight bearing and paw withdrawal threshold decrease, alterations in the tibial and femoral structures (decreased trabeculation and cortical erosions), histopathological damage (disorganized cartilage structure, hypocellularity, decreased matrix staining, disrupted tidemark integrity, synovial hyperplasia, and osteophyte formation), and changes in the astrocyte and microglia density in the lumbar spinal cord.

**Conclusion:**

SZV-1287 may have disease-modifying potential through analgesic, anti-inflammatory, and chondroprotective effects. The MIA mouse model is valuable for investigating OA-related mechanisms and testing compounds in mice at an optimal dose of 0.5 mg.

## 1 Introduction

Osteoarthritis (OA) is the most common musculoskeletal disease in humans with globally increasing prevalence and is characterized by degeneration of the weight bearing joints, such as knees and hips (Neogi and Zhang, 2013). OA mainly affects elderly people and has been reported to affect up to 50% of the population above the age of 65 years (Xing et al., 2016), meaning that nearly 300 million people are affected globally (Global and Incidence, 2018). Therefore, OA has great social, economic, and healthcare burden (Weinstein et al., 2013; Neogi, 2013; Hawker et al., 2014). The current first-line therapy for OA involves non-steroidal anti-inflammatory drugs (NSAIDs), but these are not able to improve the structural damage and often have limited systemic use owing to poor therapeutic efficacy and/or detrimental side effects (Bannuru et al., 2019). Therefore, there is an emerging need to elucidate the pain-related pathophysiological mechanisms of OA to develop effective and safe analgesics with disease-modifying potential (Kuswanto and Baker, 2024).

A novel, multitarget analgesic candidate SZV-1287 [3-(4,5-diphenyl-1,3-oxazol-2-yl) propanal oxime] is proposed to meet these criteria as it can parallelly target several disease mechanisms; this molecule was patented by us for treatment of neuropathic pain and neurogenic inflammation (patent publication number: WO/2015/159112). SZV-1287 is an oxime analog of oxaprozin, which is a widely used NSAID in the United States since 1992 for indications of OA and rheumatoid arthritis (RA) (Dallegri et al., 2005). This compound was originally formulated as a conceptually novel anti-inflammatory drug on the basis of the innovative metabolism-activated multitargeting (MAMUT) strategy. It combines two synergistic anti-inflammatory mechanisms, namely inhibition of amine oxidase copper containing 3 (AOC3)/vascular adhesion protein 1 (VAP-1) by the parent compound and cyclooxygenase (COX) by the active metabolite oxaprozin (Matyus and Chai, 2016). Our research group demonstrated that it exerts analgesic and anti-inflammatory effects in two different RA models (Horvath et al., 2017, 2018) and in traumatic (Horváth et al., 2021; Horvath et al., 2018) and diabetic (Tékus et al., 2020) neuropathy models. These effects are suggested to be mediated by reduced formation of the transient receptor potential ankyrin 1 (TRPA1) receptor activator AOC3 products (e.g., formaldehyde, methylglyoxal, hydrogen peroxide), leading to decreased nociceptor activation (Payrits et al., 2016). In addition, we showed its direct dual antagonistic effects on pain sensing TRPA1 and the closely related transient receptor potential vanilloid 1 (TRPV1) receptors (Payrits et al., 2016). In 2016, we initiated the development process for this compound with the indication of neuropathic pain and have successfully completed the phase IA clinical trial with a single ascending dosing (Horváth et al., 2021).

Well-characterized OA models with translational value are essential for developing novel analgesics and disease-modifying drugs, among which the monoiodoacetate (MIA)-induced rodent model is the most commonly used (Lampropoulou-Adamidou et al., 2014). MIA is a glyceraldehyde-3-phosphate dehydrogenase inhibitor that disrupts the glucose metabolism of the chondrocytes by causing apoptosis after intraarticular (i.a.) injection (Kalbhen, 1987). This leads to cartilage degeneration accompanied by low-grade synovitis, pathological subchondral bone remodeling, joint neovascularization, weight bearing deficit, and referred hypersensitivity on the affected hind limb that closely resembles human pathology (Combe et al., 2004). This model is particularly suitable for assessing OA-related pain behaviors (Bapat et al., 2018) because it provides joint movement changes and tactile hypersensitivity validated by pain-relieving therapies (Vonsy et al., 2009; Fernihough et al., 2004). The nocifensive behavior persists even after resolution of the acute inflammatory phase (Harvey and Dickenson, 2009), and consistent with humans, central sensitization develops (Kelly et al., 2013; McDougall et al., 2017). Broad ranges of MIA doses and experimental paradigms have been used in literature to model different components of the pathophysiological process, which make comparisons and conclusions difficult (Harvey and Dickenson, 2009; Ogbonna et al., 2013; Moilanen et al., 2015; Pitcher et al., 2016; McDougall et al., 2017). Therefore, it is important to characterize the model through complex investigational techniques related to both inflammatory and nociceptive mechanisms. Herein, we investigated the effects of our multitarget analgesic candidate SZV-1287 in the MIA-induced mouse OA model using integrated functional and morphological approaches.

## 2 Materials and methods

### 2.1 Animals

Experiments were conducted on 8–16-week-old male C57BL/6J mice weighing 20–30 g. All animals were bred and raised in the Laboratory Animal House of the Department of Pharmacology and Pharmacotherapy, Medical School, University of Pécs in cages of size 330 × 160 × 137 mm with a maximum occupancy of five mice per cage under 12-h light/dark cycles at 22°C ± 2°C and 50%–60% humidity. The mice were provided with standard mouse chow (ssniff Spezialdiäten GmbH, Soest, Germany) and water *ad libitum*. The total number of animals used in the experiments was 98, and they were randomized under the different experimental groups on the basis of the mechanonociceptive threshold of the hind paw according to the primary outcome of mechanical hyperalgesia. The number of animals per group was determined on the basis of previously reported experiments with similar complex methodologies.

### 2.2 Ethics

The animal study was conducted according to the European legislation (Directive 2010/63/EU) and Hungarian Government regulation (40/2013., II. 14.) regarding the protection of animals used for scientific purposes and was in full compliance with to the recommendations of the International Association for the Study of Pain. The study was approved by the Animal Welfare Committee of the University of Pécs and National Scientific Ethical Committee on Animal Experimentation of Hungary as well as licensed by the Government Office of Baranya County (BA02/2000–52/2018).

### 2.3 Induction of experimental OA

The right hind limbs of the mice were shaved, and ketamine (120 mg/kg) and xylazine (6 mg/kg) were administered intraperitoneally (i.p.) as anesthesia; then, 20 µL of sodium MIA solution (0.5 or 0.8 mg dissolved in saline to observe potential dose-dependent changes) was injected into the knee joint space through the patellar ligament to induce experimental OA (Horvath et al., 2016; Tékus et al., 2018). Saline at the same volume was injected into the control animals.

### 2.4 Preparation of the SZV-1287 solution

SZV-1287 was first synthesized at the Department of Organic Chemistry, Faculty of Pharmacy, Semmelweis University, Budapest, Hungary, the scaling-up process was then developed at the Institute of Organic and Medicinal Chemistry, Faculty of Pharmacy, University of Pécs, Pécs, Hungary. For this study, it was provided as previously described in the patent numbered WO/2010/029/379. The characterization data for SZV-1287, including nuclear magnetic resonance and mass spectrometry details as well as the melting point, have been published previously (Tékus et al., 2020). SZV-1287 was dissolved in a vehicle containing 20% Kolliphor HS 15 (polyethylene glycol (15)-hydroxystearate) and 80% distilled water (Horvath et al., 2018). First, Kolliphor HS 15 was heated moderately to 30°C and added to SZV-1287; the solution was then sonicated to maximize the solubility of SZV-1287, before adding distilled water (2 mg/mL) and vortexing. All solutions used in this study were always freshly prepared before injection.

### 2.5 Experimental design

The OA and non-OA mice were randomly divided into two homogenous subgroups receiving SZV-1287 i.p. or its vehicle. Groups 1 and 3 were treated with the vehicle (0.1 mL/10 g bodyweight), whereas groups 2 and 4 were administered the same volume of SZV-1287 (20 mg/kg) (Table 1). Both treatments started on day 0 approximately 20 min before i.a. MIA injection and were continued daily for 21 days. The treatments were performed every morning 20 min before the first measurements to investigate the acute-on-chronic effects of the injections. The SZV-1287 doses applied were chosen on the basis of our earlier experiments (Tábi et al., 2013; Horvath et al., 2017, 2018).

**TABLE 1:** Treatment protocols of all groups of animals involved in the study. Monoiodoacetate (MIA, 0.5 or 0.8 mg) was injected intraarticularly (i.a.) into the right knee joints of the osteoarthritic (OA) animals on day 0. Non-OA control animals received saline using the same protocol. Groups 1 and 3 were treated i.p. with the vehicle (0.1 mL/10 g body weight), whereas groups 2 and 4 were treated i.p. with the same volume of SZV-1287 (20 mg/kg).

Group		I.a. injection	Treatment	Group size
Non-OA	Group 1	Saline	Vehicle	13
	Group 2	Saline	SZV-1287	13
OA	Group 3	0.5 mg MIA	Vehicle	29
		0.8 mg MIA	Vehicle	9
	Group 4	0.5 mg MIA	SZV-1287	27
		0.8 mg MIA	SZV-1287	7

**FIGURE 1:**
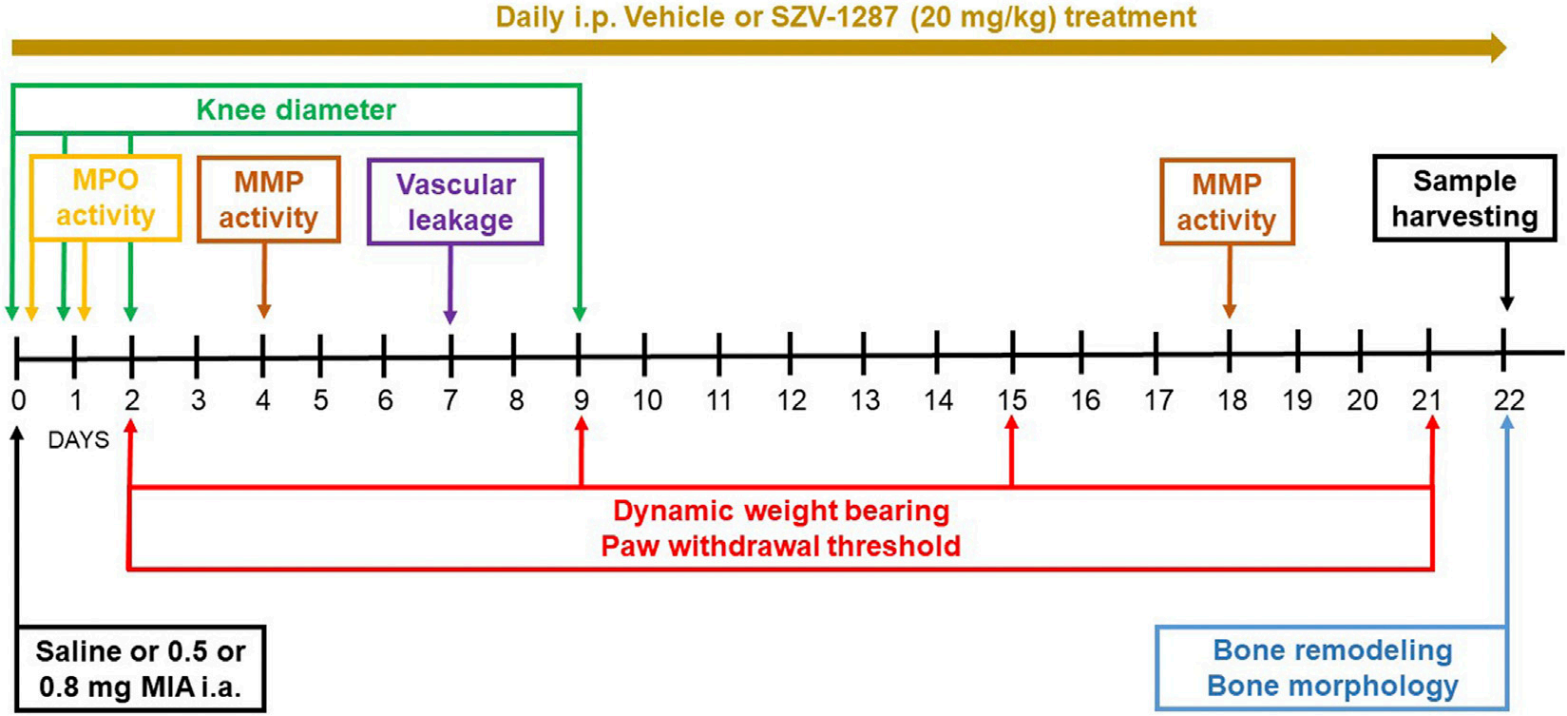
Flowchart describing the experimental design. Abbreviations: i.a., intraarticular; i.p., intraperitoneal; MMP, matrix metalloproteinase; MPO, myeloperoxidase.

The experimental design is summarized in Figure 1. The bodyweights of the mice were measured every day; knee diameter was measured on days 0 (3 and 6 h after MIA injection), 1, 2, and 9; neutrophil myeloperoxidase (MPO) activity was measured on days 0 (3 h after MIA injection) and 1; paw withdrawal threshold and dynamic weight bearing were measured on days 2, 9, 15, and 21; matrix metalloproteinase (MMP) activity was measured on days 4 and 18; vascular leakage was determined on day 7; and bone remodeling and structural bone alterations were assessed on day 22. All measurement timepoints for these complex protocols were chosen on the basis of our experience and literature data available on the different pathophysiological parameters of the model (Blom et al., 2007; Baragi et al., 2009; Walsh et al., 2007a; Xie et al., 2012). At the end of the first two experiments (on day 21), the mice were euthanized with sodium pentobarbital (100 mg/kg, i.p.), followed by micro-computed tomography (micro-CT) analysis and harvesting of the knee joints for histopathological evaluations. At the end of the third experiment (on day 21), the animals were deeply anesthetized using sodium pentobarbital (70 mg/kg, i.p.) for bone remodeling measurements and were transcardially perfused with phosphate-buffered saline (PBS) followed by 4% paraformaldehyde. The brains and spinal cords of the animals were then removed for immunohistochemical evaluation of the astrocytes and microglia activation.

All treatments, measurements, and data analyses were performed in a blinded manner, and the animals were coded throughout the experiments. The codes were revealed only after the principal investigator completed all the evaluations (Á.I.H). Each experimental group was represented in all studies to minimize the risk of confounding factors, such as unexpected noise, smell, weather changes, and temperature. Each investigational technique and treatment were performed by the same researcher/technician throughout the study.

### 2.6 Determination of dynamic weight bearing on the hind limbs

Weight distribution decrease on the hind limbs as an indicator of spontaneous pain was assessed using an advanced dynamic weight bearing apparatus (Bioseb, Vitrolles, France) (Tékus et al., 2018). The mice were placed in a perspex chamber with a pressure-sensitive floor and were allowed to move freely. The weights borne on the hind limbs (measured in grams) were tracked and recorded over a 5-min period. Following analysis of the recordings, weight bearing was calculated as the ratio of weight borne on one hind limb and the total weight borne by the hind limbs (i.e., [weight borne on the ipsilateral or contralateral hind limb/(weight borne on the ipsilateral hind limb + weight borne on the contralateral hind limb)] × 100).

### 2.7 Determination of paw withdrawal threshold

Paw withdrawal threshold decrease (mechanical hyperalgesia) was assessed as an indicator of referred pain through dynamic plantar esthesiometry (Ugo Basile, Gemonio, Italy) (Horvath et al., 2016). Mice were placed in plexiglass boxes with metal mesh floors so that they could move freely. Following 30 min of acclimation, the plantar surfaces of the hind paws were touched using a straight metal filament with a lifting force of 2.5 g/s until the mice retracted their paws (with the cutoff value being 10 g). The mean of three measurements was recorded as the withdrawal threshold from both hind paws and was expressed in grams. The percentage change of the withdrawal threshold was calculated for comparison with the mean initial baseline value.

### 2.8 Determination of knee diameter

The knee diameters were measured with a digital caliper (OWIM GmbH and Co. KG, Neckarsulm, Germany) (Horvath et al., 2016). Each mouse was stably handled by one investigator, while another person extended one of the hind limbs to measure the knee joint thickness (expressed in millimeters) in both the anteroposterior and mediolateral directions (Botz et al., 2013).

### 2.9 Functional *in vivo* optical imaging

Neutrophil MPO activity, MMP activity, vascular leakage, and bone remodeling in the knee joint were determined using the IVIS^®^ Lumina III *in vivo* imaging system (PerkinElmer, Waltham, MA, USA) (Horváth et al., 2018). One day before the measurements, hair was removed from both hind limbs through shaving and application of Veet^®^ cream in ketamine (120 mg/kg, i.p.) and xylazine (6 mg/kg, i.p.) anesthesia to prevent scattering/absorption of the light signal. Neutrophil MPO activity was measured using luminol (5-amino-2,3-dihydro-1,4-phthalazine-dione) sodium salt injection (150 mg/kg, i.p.) dissolved in saline (30 mg/mL); MMP activity was measured by an activatable fluorescent probe for MMP-2, -3, -7, -9, -12, and -13 (MMPSense 750 FAST; 2 nmol/subject, i.v.); vascular leakage was measured with the vascular fluorescent probe AngioSense 750 EX (2 nmol/subject, i.v.), and bone remodeling was measured using the targeted fluorescent probe OsteoSense 680 EX (2 nmol/subject, i.v.). Injections of the probes and measurements were performed under ketamine (120 mg/kg, i.p.) and xylazine (6 mg/kg, i.p.) anesthesia. Bioluminescence imaging was performed 10 min after injection (120 s acquisition, Binning = 8, F/stop = 1), and fluorescent imaging was performed 24 h post injection (auto acquisition time, Binning = 8, F/stop = 2, excitation/emission filters: 740/790 nm for MMP activity and vascular leakage or 680/845 nm for bone remodeling). Using Living Image^®^ software (PerkinElmer, Waltham, MA, USA) identical regions of interest (ROIs) were applied around the knee joints, and luminescence was expressed as the total radiance (total photon flux/s) while fluorescence was expressed as the total radiant efficiency ([photons/s/cm^2^/sr]/[µW/cm^2^]) within the ROI.

### 2.10 Assessment of bone morphology in the knee joint

Bone morphology in the knee joint was assessed through the SkyScan 1,176 *in vivo* micro-CT (Bruker, Kontich, Belgium) (Tékus et al., 2018). On the last day of the experiment (day 21), the mice were anesthetized using sodium pentobarbital (70 mg/kg, i.p.), and the periarticular regions of the tibia and femur were repeatedly scanned with a voxel size of 17.5 μm. The scans were reconstructed, and the bone structures were assessed using the CT Analyser^®^ software. Standardized ROIs were drawn around the entire epiphysis of the distal femur or proximal tibia, from which bone volume density (bone volume/total volume), trabecular number (Tb.N), trabecular separation (Tb.Sp), trabecular pattern factor (Tb.Pf), volume of open pore space (Po.V (op)), and open porosity (Po(op)) were quantified.

### 2.11 Histopathological evaluation of the knee joints

On the last day of the experiment (day 21), the knee joints of the anesthetized mice were harvested and post-fixed in 4% formaldehyde for 8 h. Then, the samples were decalcified in a demineralizing solution containing 7% (w/v) AlCl_3_, 5% (v/v) formic acid, and 8.5% (v/v) HCl for 8 h at 4°C, washed in Sörensen phosphate buffer and dehydrated at 4°C for 8 h in 5% (w/v) in saccharose, followed by successive immersion in 10% and 15% (w/v) saccharose for 8 h each. The samples were embedded in paraffin, sliced into sections (3–5 μM), and stained with Safranin O. The histopathological changes to the knee joints were semiquantitatively determined using the modified Mankin scoring system and some additional parameters by an investigator who was blinded to the study (Horvath et al., 2016). The structure, cellular abnormalities, and matrix staining were assessed through the modified Mankin score on scales of 0–6, 0–3, and 0–4, respectively. Additionally, the tidemark integrity (0–1), synovial hyperplasia and inflammatory cell infiltration (0–3), as well as osteophyte formation (0–1) were scored. The scores for the six criteria were accumulated to generate a composite score with values ranging from 0 to 18.

### 2.12 Determination of glial cell immunoreactivity in central nervous system (CNS) areas related to pain transmission

#### 2.12.1 Tissue processing for glial cell detection

At the end of the study, the mice were deeply anesthetized with sodium pentobarbital (70 mg/kg, i.p.) and transcardially perfused with PBS followed by 4% paraformaldehyde. The brains as well as the L3–L6 spinal cord segments innervating both the knee joints and hind paws were removed and post-fixed for 4 h in the same fixative before transferring into a medium containing 30% sucrose dissolved in 0.1 M PBS at 4°C until they sank for cryoprotection (at least overnight). Free floating sections (30 µm) were prepared on a freezing microtome (Leica Biosystems, Nussloch, Germany) and stored at −20°C in antifreeze solution until further processing.

#### 2.12.2 Glial fibrillary acidic protein (GFAP) and ionized calcium-binding adaptor molecule 1 (Iba1) immunostaining of the dorsal horn of the spinal cord (SC), periaqueductal gray matter (PAG), and somatosensory cortex (SSC)

GFAP and Iba1 labeling were used in the SC, PAG, and SSC to visualize the astrocytes (Eng et al., 2000) and activated microglia (Ito et al., 1998), respectively. In brief, free-floating serial SC sections were washed in 0.05% TRIS-buffered saline (TBS, pH: 7.6) before treating with methanol and 3% H_2_O_2_ for 30 min; the PAG and SSC sections were washed in 0.1 M PBS and treated with only 3% H_2_O_2_ for 30 min. Next, the SC sections were thoroughly washed in TBS, while the PAG and SSC sections were washed in PBS; then, non-specific binding was prevented by incubating the sections for 1 h in 3% bovine serum albumin (BSA) in TBS containing 0.5% Triton X-100 (for SC) or in 3% normal goat serum (NGS) in PBS containing 0.5% Triton X-100 (for PAG and SSC). Subsequently, the SC sections were repeatedly rinsed in TBS while the PAG and SSC sections were rinsed in PBS for 1 h before incubation for one night at 4°C with a monoclonal mouse anti-GFAP antibody (1:5,000) or with a polyclonal rabbit anti-Iba1 antibody (1:10,000). After thorough rinsing, the sections were incubated with anti-rabbit or anti-mouse biotinylated secondary antibodies (1:200) for 2 h before being washed and incubated in avidin-biotin-horseradish peroxidase (1:100 for SC or 1:200 for PAG and SSC) for 2 h. The labeled cells were visualized in 0.025% of 3,30-diaminobenzidine and 0.01% of H_2_O_2_ in TBS (for SC) or in PBS (for PAG and SSC) for 3 min. Lastly, the sections were mounted, dried overnight, dehydrated in graded alcohol, cleared, and coverslipped with Eukit. The slides were coded before quantification to ensure objectivity. The images were acquired on a Nikon Eclipse Ti-U workstation.

#### 2.12.3 Cell quantification

All of the immunolabeled cells were counted manually. A single experimenter who was blinded to the group identification of each animal performed the data collection. The code was not broken until the cell counting analyses were completed.

Imaging analyses were performed using a Nikon Eclipse Ni-E bright-field microscope equipped with a computer-controlled stage operated using Neurolucida software (v07, MBF Bioscience, Williston, VT, USA), and quantitative analyses were carried out using a modified unbiased stereology protocol that has been reported to successfully quantify GFAP and Iba1 neurons in the PAG, SSC, and SC (Stornetta et al., 2002). GFAP or Iba1 immunoreactive cells were counted in three CNS regions known to play crucial roles in pain perception and processing: a standardized area of the Rexed laminae I-II in the dorsal horn of the L3–L6 segments of the SC, PAG (approximately 3.8–4.24 mm caudal to the bregma), and SSC representation of the hind limb (approximately 0.46–0.7 mm caudal to the bregma). The planes of the sections were standardized using the Atlas of Paxinos and Franklin (Paxinos and Franklin, 2001). All the labeled astrocytes and microglial cells were counted within the borders using an objective of ×10 or ×20 magnification. The numbers of GFAP and Iba1 immunopositive cells were counted in the areas of interest and expressed as number of cells per mm^2^. For the statistical analyses, the averages of 2–4 sections of 30 µm thickness were used from each of the animals.

### 2.13 Data and statistical analyses

The data were expressed as means with 95% confidence intervals (CIs) and analyzed using GraphPad Prism 8 software (GraphPad Software, San Diego, CA, USA). In the case of normal distribution, parametric tests were performed except for the histopathological scores. Weight bearing asymmetry and knee edema were evaluated using the mixed-effects model followed by Dunnet’s, Sidak’s, or Tukey’s multiple comparisons test; the paw withdrawal threshold decrease (mechanical hyperalgesia) was assessed by two-way repeated measures analysis of variance (ANOVA) followed by Sidak’s multiple comparisons test; the neutrophil MPO and MMP activity increases, vascular leakage, bone remodeling, structural bone alterations, and glial cell immunohistochemistry were assessed with two-way ANOVA followed by Sidak’s multiple comparisons test; the histopathological alterations were analyzed using Kruskal–Wallis test followed by Dunn’s multiple comparisons test. For the parametric tests, the ANOVA tables are shown in the Supplementary Materials (Supplementary Tables S1-S3). Since statistical significance and non-significance are not evidence of biological relevance and equivalence, respectively, the effect sizes were also determined using Hedges’ g (difference in means divided by the pooled and weighted standard deviation) in all cases. An effect size >0.2 was assumed as small, >0.5 was considered as medium, and >0.8 was designated as large (Cohen, 1992).

### 2.14 Materials

Ketamine (Calypsol^®^) was purchased from Gedeon Richter Plc. (Budapest, Hungary); xylazine (Sedaxylan^®^) was obtained from Eurovet Animal Health B.V. (Bladel, Netherlands); sodium MIA, Kolliphor HS 15, and 3,30-diaminobenzidine were sourced from Sigma-Aldrich (St. Louis, MO, USA); sodium pentobarbital (Euthanimal^®^) was procured from Alfasan Nederland B.V. (Woerden, Netherlands); Veet^®^ cream was acquired from Reckitt (Slough, United Kingdom); luminol sodium salt was purchased from Gold Biotechnology (Olivette, MO, USA); MMPSense 750 FAST, AngioSense 750 EX, and OsteoSense 680 EX were from PerkinElmer (Waltham, USA); BSA, NGS, anti-rabbit and anti-mouse biotinylated secondary antibodies, and avidin-biotin-horseradish peroxidase (Vectastain Elite ABC Kit) were supplied by Vector Laboratories (Newark, CA, USA); monoclonal mouse anti-GFAP antibody was sourced from Cell Signaling Technology (Danvers, MA, USA, Cat# 3670S, RRID: AB_561049); and polyclonal rabbit anti-Iba1 antibody was obtained from FUJIFILM Wako Chemicals Europe GmbH (Neuss, Germany, Cat# 019-19741, RRID: AB_839504).

## 3 Results

### 3.1 SZV-1287 inhibits MIA-induced spontaneous pain behavior and mechanical hyperalgesia

**FIGURE 2:**
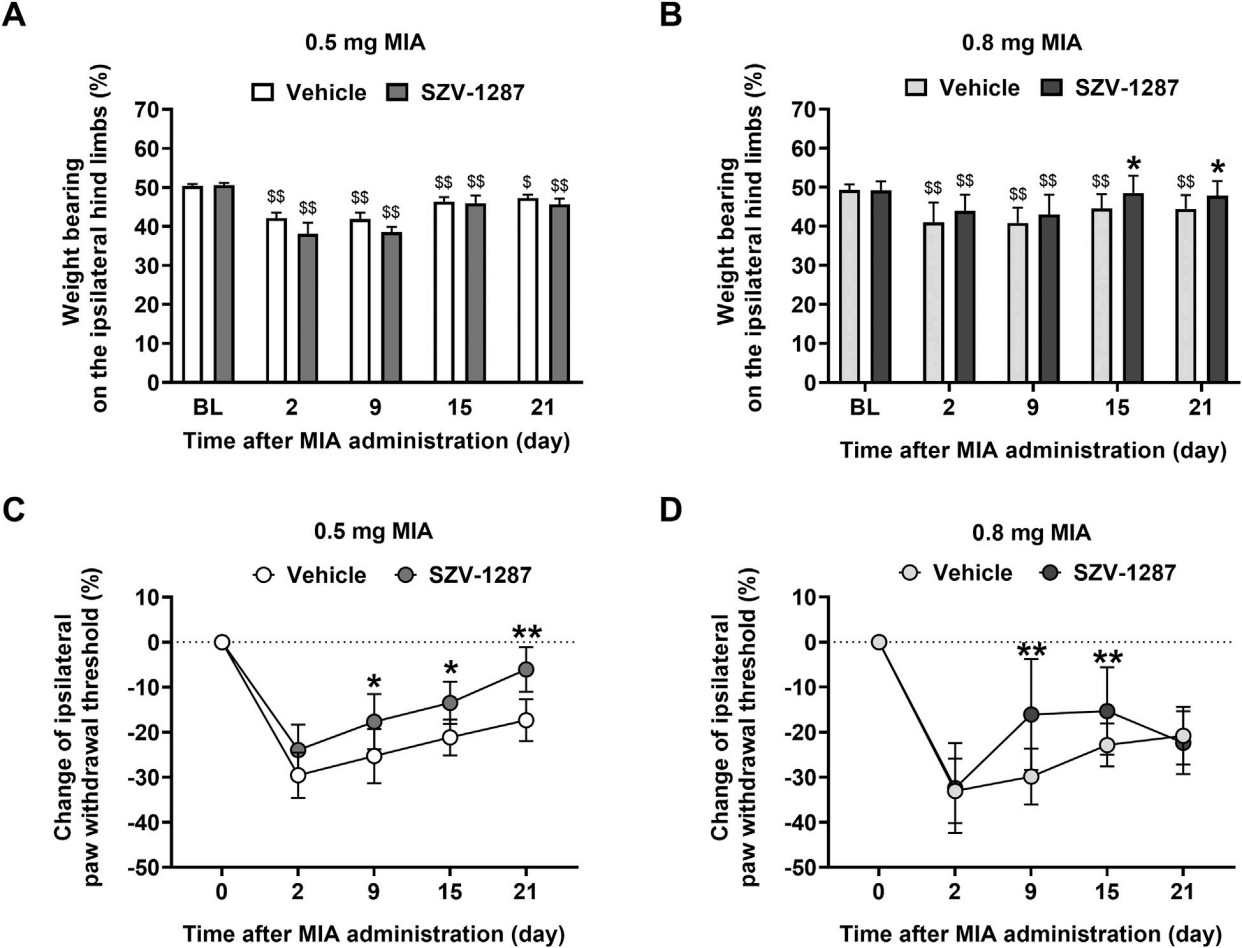
Effects of SZV-1287 on MIA-induced weight bearing asymmetry and mechanical hyperalgesia. Dynamic weight-bearing and percentage changes of the withdrawal thresholds of the ipsilateral hind limbs of the **(A,C)** 0.5- and **(B,D)** 0.8-mg MIA-injected SZV-1287-treated (20 mg/kg i.p. every day during the 21-day experimental period) mice compared to the respective vehicle-treated groups. The data are shown as means with 95% CI of n = 7–29 mice/group, ^$$^ *g* > 0.8 vs. respective baseline (BL), *g > 0.5, **g > 0.8 vs. respective vehicle-treated group. *G* indicates the effect size calculated by Hedges’ g.

On day 2, doses of 0.5 and 0.8 mg of MIA were administered to induce weight bearing deficit on the ipsilateral hind limbs of vehicle-treated mice with large effect sizes compared to the respective baseline values, which were maintained throughout the 21 days of the study (Figures 2A,B; Supplementary Table S4). There were no differences between the effects of the two doses at any timepoint (Supplementary Figure S1A; Supplementary Table S4). Although saline injection impaired weight bearing slightly and transiently compared to the baseline and the contralateral side, presumably resulting from the injection process (Supplementary Figure S1A, B; Supplementary Tables S4 and S5), the effects of MIA were greater (Supplementary Figure S1A–C; Supplementary Tables S4 and S5). In the late phase of the model (on days 15 and 21), when MIA-induced cartilage loss was expressed more, the weight bearing asymmetry was maintained in both MIA-injected groups but not in the saline-injected subjects (Supplementary Figures S1A, C, D; Supplementary Tables S4 and S5). In this phase, the 0.8 mg dose of MIA did not induce spontaneous pain behavior in the SZV-1287-treated group (Figure 2B; Supplementary Figure S1F; Supplementary Tables S5 and S6). Weight bearing on the ipsilateral hind limbs was higher in the 0.8 mg MIA-injected and SZV-1287-treated group from day 15 compared to the vehicle-treated group (Figure 2B; Supplementary Table S6).

Both doses of MIA, but not the saline treatment, induced approximately 30% decrease in the withdrawal threshold of the ipsilateral hind paws of the vehicle-treated mice with large effect sizes 2 days after the intraarticular injections (Figures 2C,D; Supplementary Tables S7 and S8), which gradually decreased to 20% by the end of the study. Similar to the MIA-induced weight bearing asymmetry, mechanical hyperalgesia was not dependent on the dose of MIA used (Supplementary Figure S1G; Supplementary Table S7). No considerable nociceptive changes were detected on the contralateral hind paws (Supplementary Figure S1H; Supplementary Table S9). Mechanical hyperalgesia was milder in the 0.5 mg MIA-injected and SZV-1287-treated group from day 15 compared to the vehicle-treated group (Figure 2C; Supplementary Table S10). In the 0.8 mg MIA-injected group, SZV-1287 decreased mechanical hyperalgesia with large effect sizes on days 9 and 15, when the inflammatory phase switched to non-inflammatory phase (Figure 2D; Supplementary Table S10). The lack of an inhibitory effect on day 21 is likely owing to the more robust cartilage destruction in response to the higher MIA dose causing increased production of inflammatory mediators and tissue injury, which could be less sensitive to the inhibitory abilities of 20 mg/kg dose of SZV-1287.

### 3.2 SZV-1287 reduces MIA-induced edema of the knee

Both MIA doses (and not saline treatment) induced approximately 30% anteroposterior and mediolateral knee diameter increases in the vehicle-treated mice with large effect sizes peaking at 6 h (Supplementary Figure S2A; Supplementary Tables S11–S14). After 6 h, the knee diameter decreased gradually and reached the baseline value for day 9. Similar to the pain-related parameters, knee edema was also independent of the MIA dose (Supplementary Figures S2A, B; Supplementary Tables S11–S14). SZV-1287 treatment reduced the 0.8 mg MIA-induced anteroposterior (at 6 h) and mediolateral knee diameter increases (at 3 and 6 h on day 2) with medium effect sizes (Supplementary Figure S2B; Supplementary Tables S11, S12, S15, S16).

### 3.3 SZV-1287 inhibits MIA-induced histopathological damage of the knee

**FIGURE 3:**
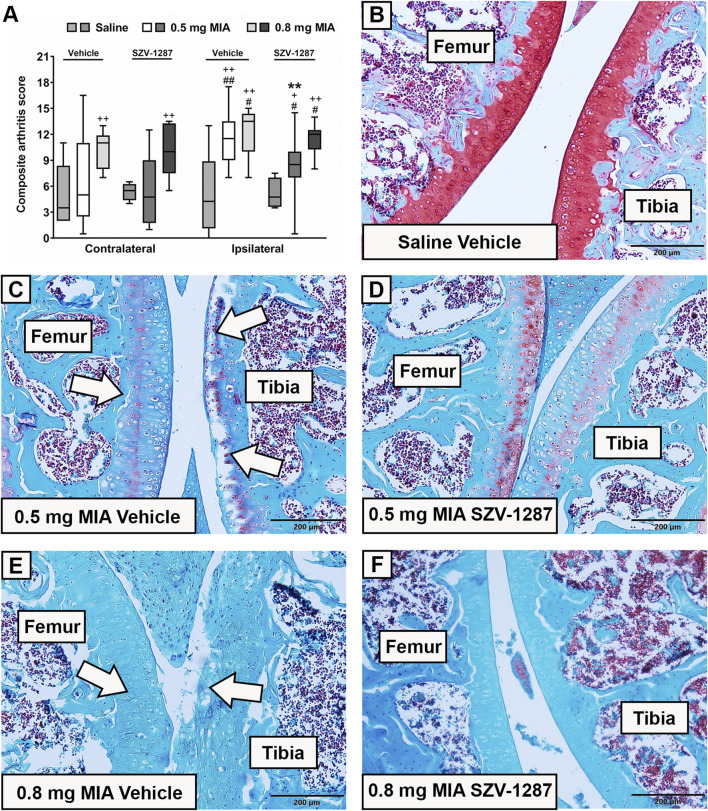
Effects of SZV-1287 on MIA-induced histopathological alterations. **(A)** Semiquantitative scoring of the histopathological alterations in the contralateral and ipsilateral knee joints of SZV-1287-treated (20 mg/kg i.p. every day during the 21-day experimental period) MIA-injected mice compared to the vehicle-treated groups on day 22 and **(B–F)** representative slides of the ipsilateral knee joints stained with Safranin O (× 10 magnification). White arrows show the most characteristic MIA-induced histopathological alterations of the cartilage layer, including structural disorganization, hypocellularity, reduced matrix staining, and disrupted tidemark integrity. Box plots represent medians of the composite scores for n = 6–15 mice/group, ^#^ *g* > 0.5, ^##^ *g* > 0.8 vs. respective contralateral side, ^+^ *g* > 0.5, ^++^ *g* > 0.8 vs. respective saline-injected group, **g > 0.8 vs. respective vehicle-treated group. *G* indicates the effect size calculated by Hedges’ g**.**

Both 0.5 and 0.8 mg doses of MIA induced histopathological alterations, such as surface irregularities, structural disorganization, hypocellularity, and reduced matrix staining, of the cartilage layer as well as synovial hyperplasia, often disrupted tidemark integrity, and osteophyte formation in the ipsilateral knee joints of the vehicle-treated mice. These changes resulted in higher composite arthritis scores compared to the saline-injected mice and compared to the contralateral knee joints (Figure 3; Supplementary Tables S17 and S18). The composite arthritis scores of the ipsilateral knees showed no considerable differences between the two MIA doses (Figure 3A; Supplementary Table S17). The most prominent microscopic alteration in response to 0.8 mg of MIA was the total loss of cartilage matrix staining (Figures 3E,F), which was observed in both the contralateral and ipsilateral knee joints. Interestingly, moderate alterations were also detected in the ipsilateral knee joints of the saline-injected mice and in the contralateral knee joints of the MIA-injected mice (Figure 3A; Supplementary Table S17), which could be explained by mechanical injury caused by the intraarticular injection and overload of the contralateral hind limbs, respectively. SZV-1287 treatment reduced the histopathological damage of the group receiving 0.5 mg of MIA with a large effect size but not for the 0.8 mg MIA-induced group (Figure 3; Supplementary Table S17).

### 3.4 SZV-1287 decreases MIA-induced neutrophil MPO activity increase

*In vivo* imaging was performed only for the 0.5 mg MIA group to obtain information on the inflammatory molecular mechanisms and microarchitectural changes since no differences were observed in any of the functional and morphological parameters between the two doses. In addition, 30% mortality occurred in the case of the group receiving 0.8 mg of MIA.

**FIGURE 4:**
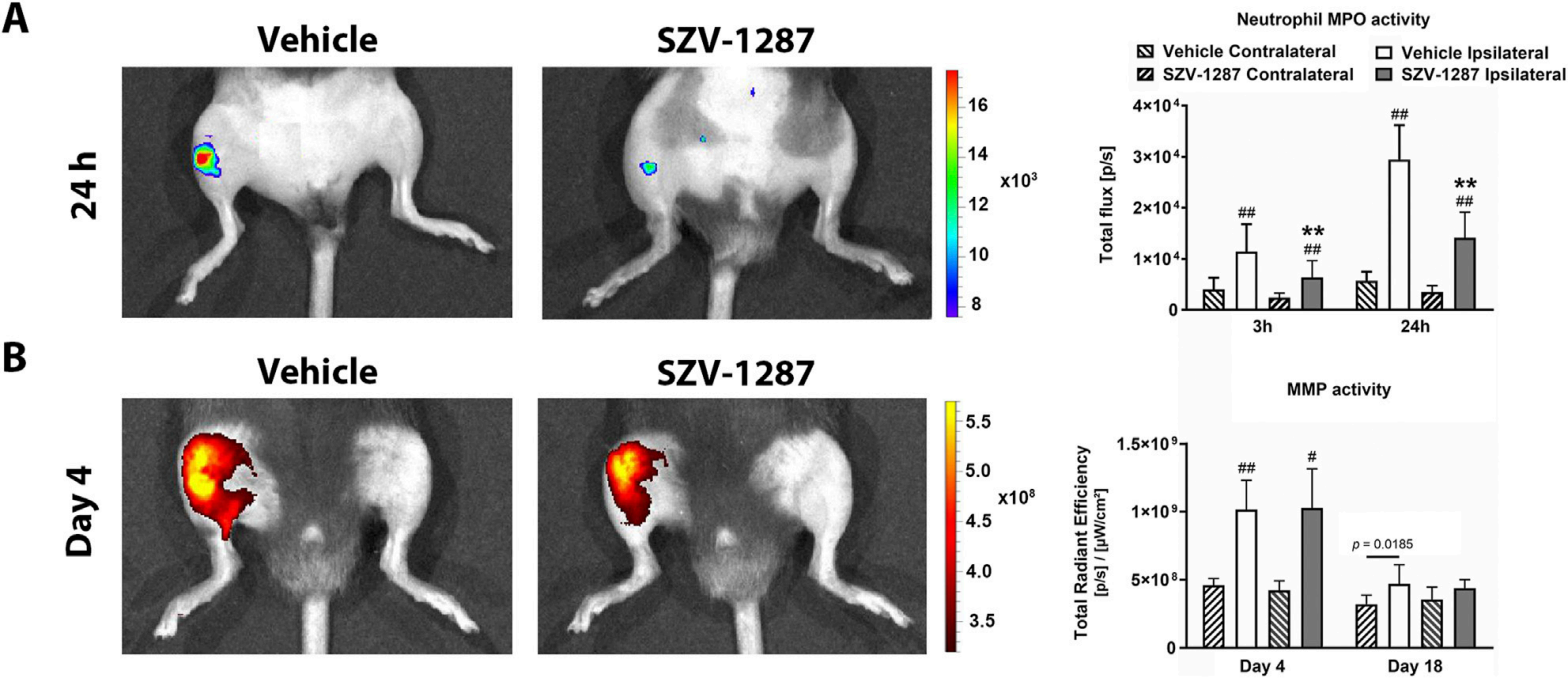
Effects of SZV-1287 on MIA-induced neutrophil myeloperoxidase (MPO) and matrix metalloproteinase (MMP) activity increases. Representative images and quantitative analyses of the **(A)** neutrophil MPO and **(B)** MMP activities in the ipsilateral knee joints of SZV-1287-treated (20 mg/kg i.p. every day during the 21-day experimental period) 0.5-mg MIA-injected mice compared to the vehicle-treated group after 3 and 24 h, as well as 4 and 18 days after osteoarthritis induction, respectively. The data are shown as means with 95% CI of n = 6–12 mice/group in the case of neutrophil MPO activity and n = 5–6 mice/group in the case of MMP activity, ^#^g > 0.5, ^##^ *g* > 0.8 vs. respective contralateral side, **g > 0.8 vs. vehicle-treated group. *G* indicates the effect size calculated by Hedges’ g.

**FIGURE 5:**
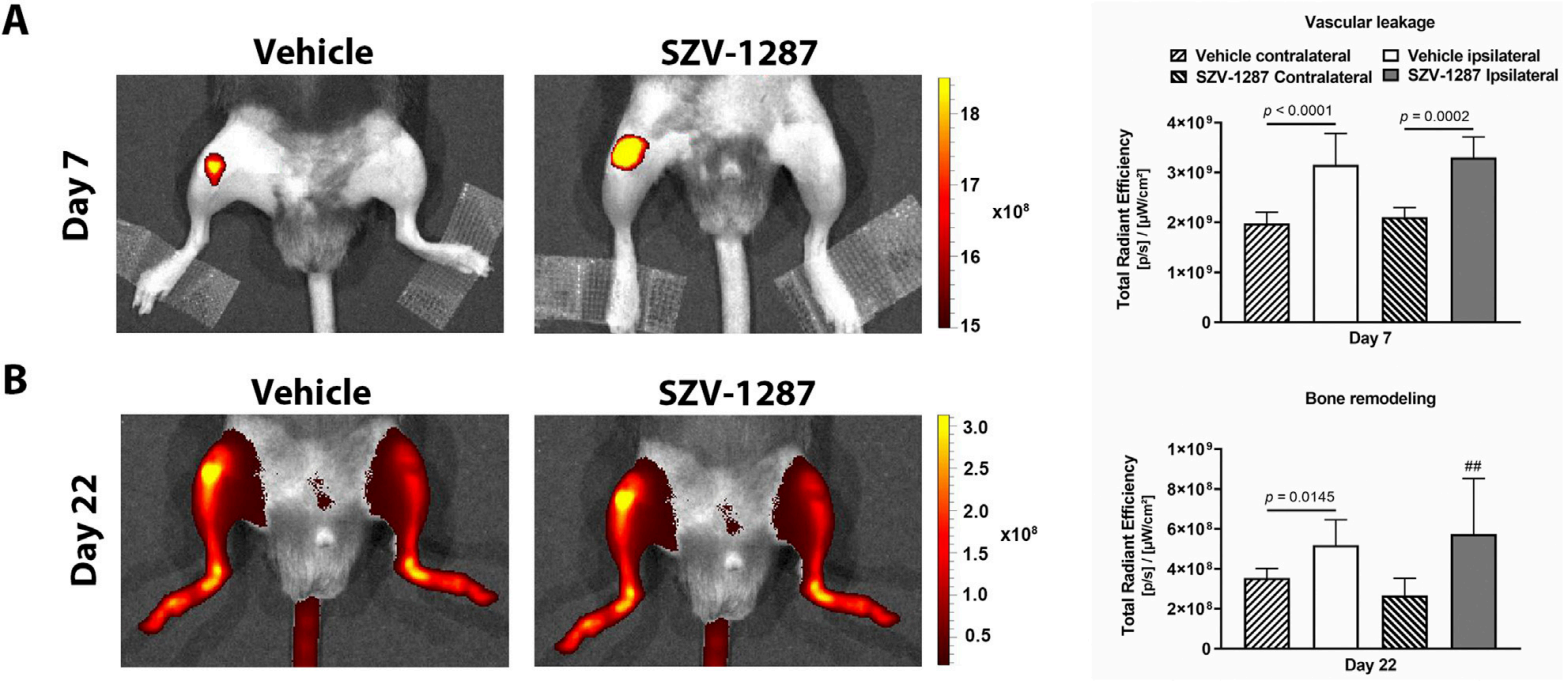
Effects of SZV-1287 on MIA-induced vascular leakage and bone remodeling. Representative images and quantitative analyses of **(A)** vascular leakage and **(B)** bone remodeling in the ipsilateral knee joints of the SZV-1287-treated (20 mg/kg i.p. every day during the 21-day experimental period) 0.5-mg MIA-injected mice compared to the vehicle-treated group 7 days and 22 days after osteoarthritis induction, respectively. The data are shown as means with 95% CI of n = 5–6 mice/group in the case of vascular leakage and n = 3–8 mice/group in the case of bone remodeling, ^##^ *g* > 0.8 vs. respective contralateral side. *G* indicates the effect size calculated by Hedges’ g.

Luminol-derived bioluminescence showed neutrophil MPO activity increases in the ipsilateral knee joints of the vehicle-treated mice with large effect sizes compared to the contralateral sides at 3 h, which further increased at 24 h (Figure 4A; Supplementary Table S19). MMP-activity-related fluorescent signals increased with large effect sizes in the same regions on day 4 and decreased thereafter, but remained elevated on day 18 (Figure 4B; Supplementary Table S19). Enhanced fluorescence signals reflecting vascular leakage caused by inflammatory vascular changes, such as hyperpermeability and neovascularization, as well as intensive bone remodeling were also observed in response to MIA on days 7 (Figure 5A) and 22 (Figure 5B), respectively, but these differences showed small effect sizes (Supplementary Table S19). SZV-1287 treatment reduced the MIA-induced neutrophil MPO activity increase with large effect sizes at both 3 and 24 h (Figure 4A; Supplementary Table S20) but did not substantially affect the other functional *in vivo* optical imaging parameters (Figures 4B, 5; Supplementary Table S20).

### 3.5 SZV-1287 does not alter MIA-induced microarchitectural changes of the bones

**FIGURE 6:**
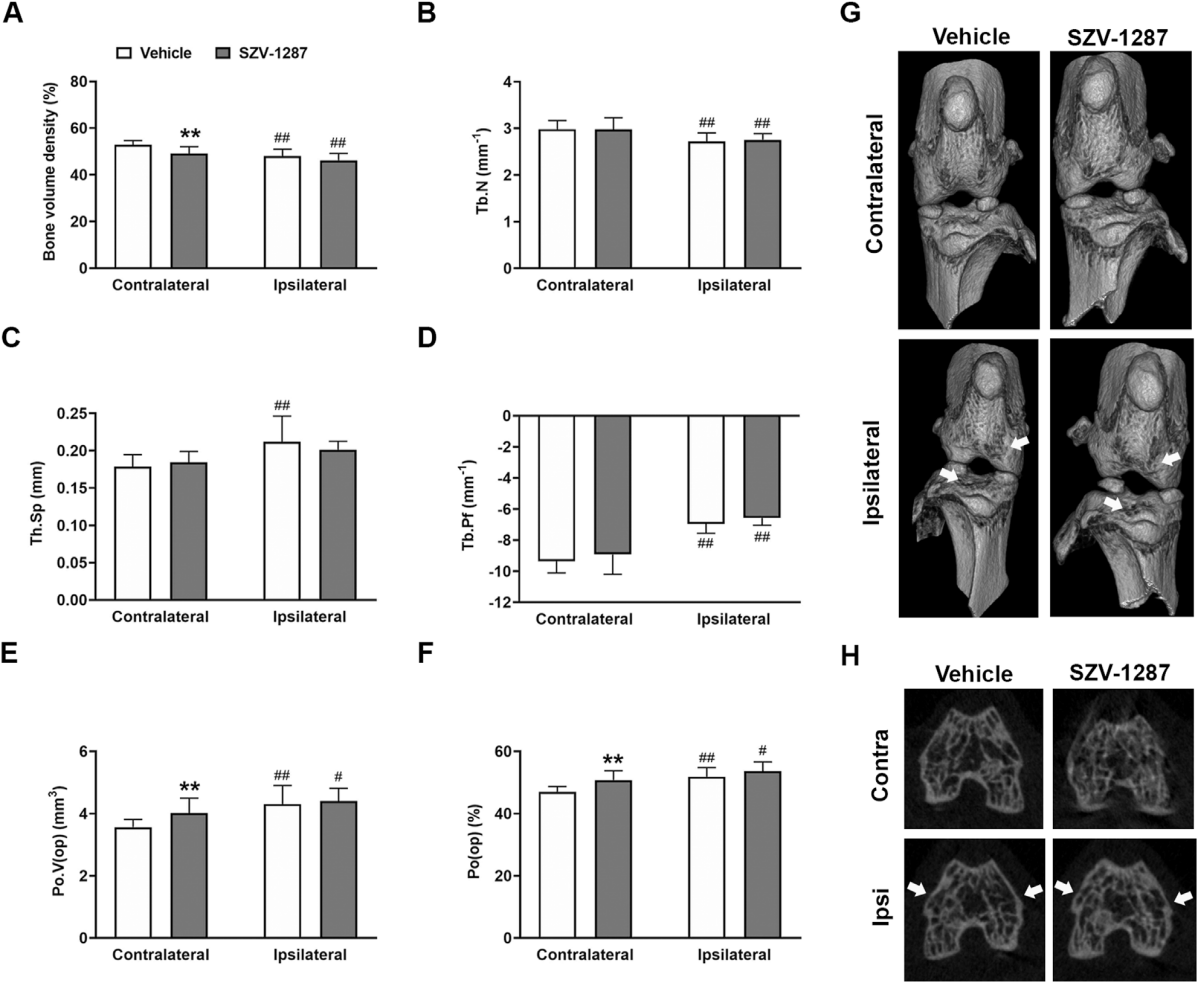
Effects of SZV-1287 on MIA-induced microarchitectural changes of the distal femur. Quantifications of **(A)** bone volume density, **(B)** trabecular number (Tb.N), **(C)** trabecular separation (Tb.Sp), **(D)** trabecular pattern factor (Tb.Pf), **(E)** volume of open pore space (Po.V (op)), and **(F)** open porosity (Po(op)) in contralateral and ipsilateral femoral epiphyses of the SZV-1287-treated (20 mg/kg i.p. every day during the 21-day experimental period) 0.5-mg MIA-injected mice compared to the vehicle-treated group on day 22. **(G)** Representative 3D reconstructions of the contralateral and ipsilateral knee joints and **(H)** axial CT slices demonstrating cortical bone loss and osteophyte formation (cortical irregularities and osteophytes highlighted by arrowheads). The data are shown as means with 95% CI of n = 8–10 mice/group, ^#^ *g* > 0.5, ^##^ *g* > 0.8 vs. respective contralateral side, **g > 0.8 vs. vehicle-treated group. *G* indicates the effect size calculated by Hedges’ g.

The most prominent MIA-induced alterations of the subchondral trabecular bone microarchitecture were bone resorption (indicated by decreased volume density and trabecular number, as well as increased trabecular separation and pattern factor), and cortical erosions (indicated by increased open pore space volume and open porosity compared to the contralateral side) (Figures 6A–F. Supplementary Figure S3A). All these parameters changed with large effect sizes in the distal femur and with medium effect sizes in the proximal tibia, except for trabecular separation and open porosity that remained unchanged in the proximal tibia (Supplementary Tables S21 and S22). Cortical erosions and osteophyte formation were also visually apparent on the 3D reconstructions (Figure 6G) and axial CT slices (Figure 6H), respectively. SZV-1287 treatment did not substantially affect the bone microarchitectural changes of the ipsilateral knee compared to the vehicle-treated group, but it caused minimal alterations to the contralateral side (Figures 6A–F; Supplementary Figure S3A).

### 3.6 SZV-1287 inhibits MIA-induced astrocyte and microglia density increases in the spinal dorsal horn

**FIGURE 7:**
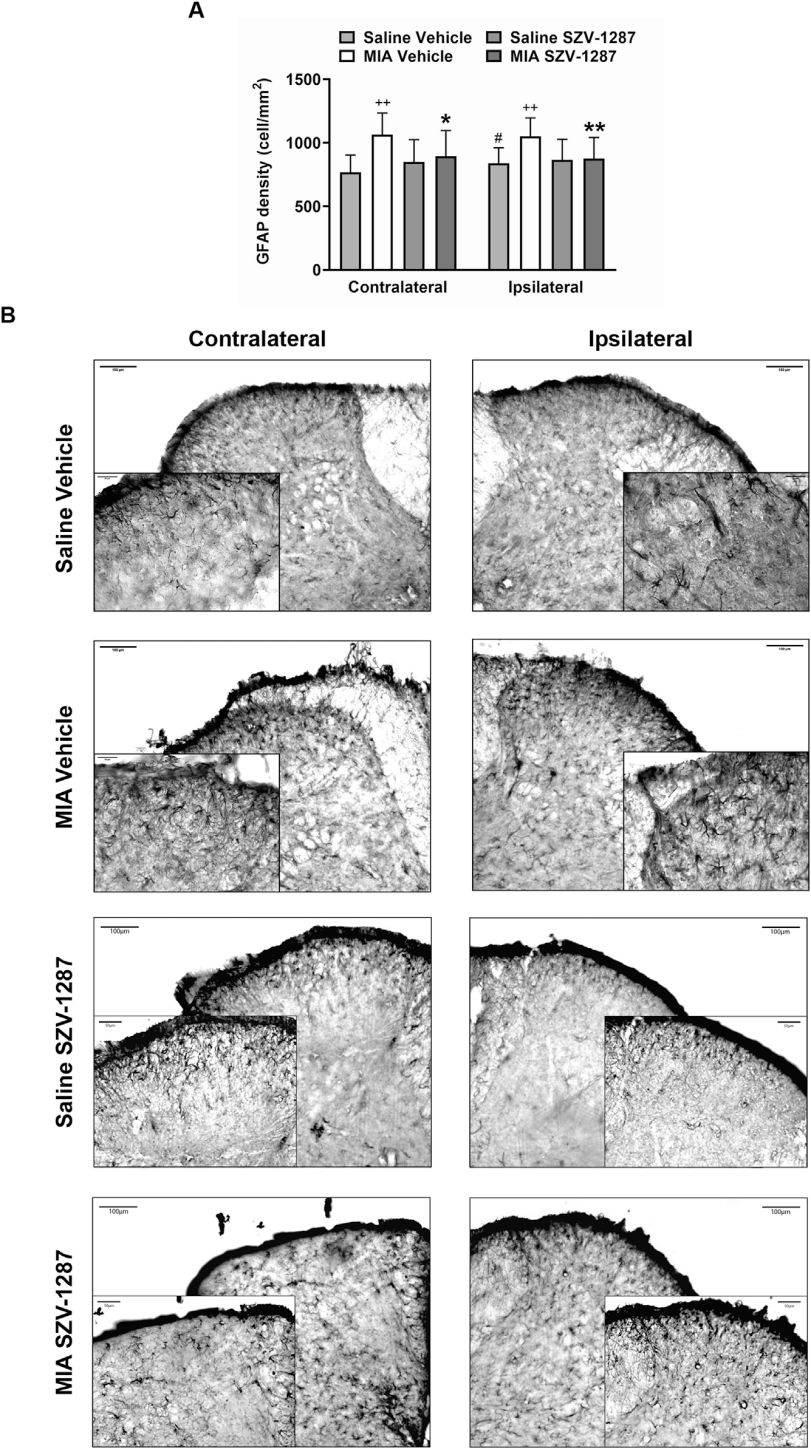
Effect of SZV-1287 on MIA-induced astrocyte density increase in the lumbar region of the spinal dorsal horn. **(A)** Quantitative analysis and **(B)** representative images of the astrocyte marker glial fibrillary acidic protein (GFAP) immunoreactivity (× 10 magnification) in the contralateral and ipsilateral L3–L6 spinal dorsal horn of the SZV-1287-treated (20 mg/kg i.p. every day during the 21-day experimental period) 0.5-mg MIA-injected mice compared to the vehicle-treated group on day 22. Data are shown as means with 95% CI of n = 5–8 mice/group, 2–4 sections/mouse, **g > 0.8 vs. respective saline-injected group, ^#^ *g* > 0.5 vs. respective contralateral side, *g > 0.5, **g > 0.8 vs. vehicle-treated group. *G* indicates the effect size calculated by Hedges’ g.

**FIGURE 8:**
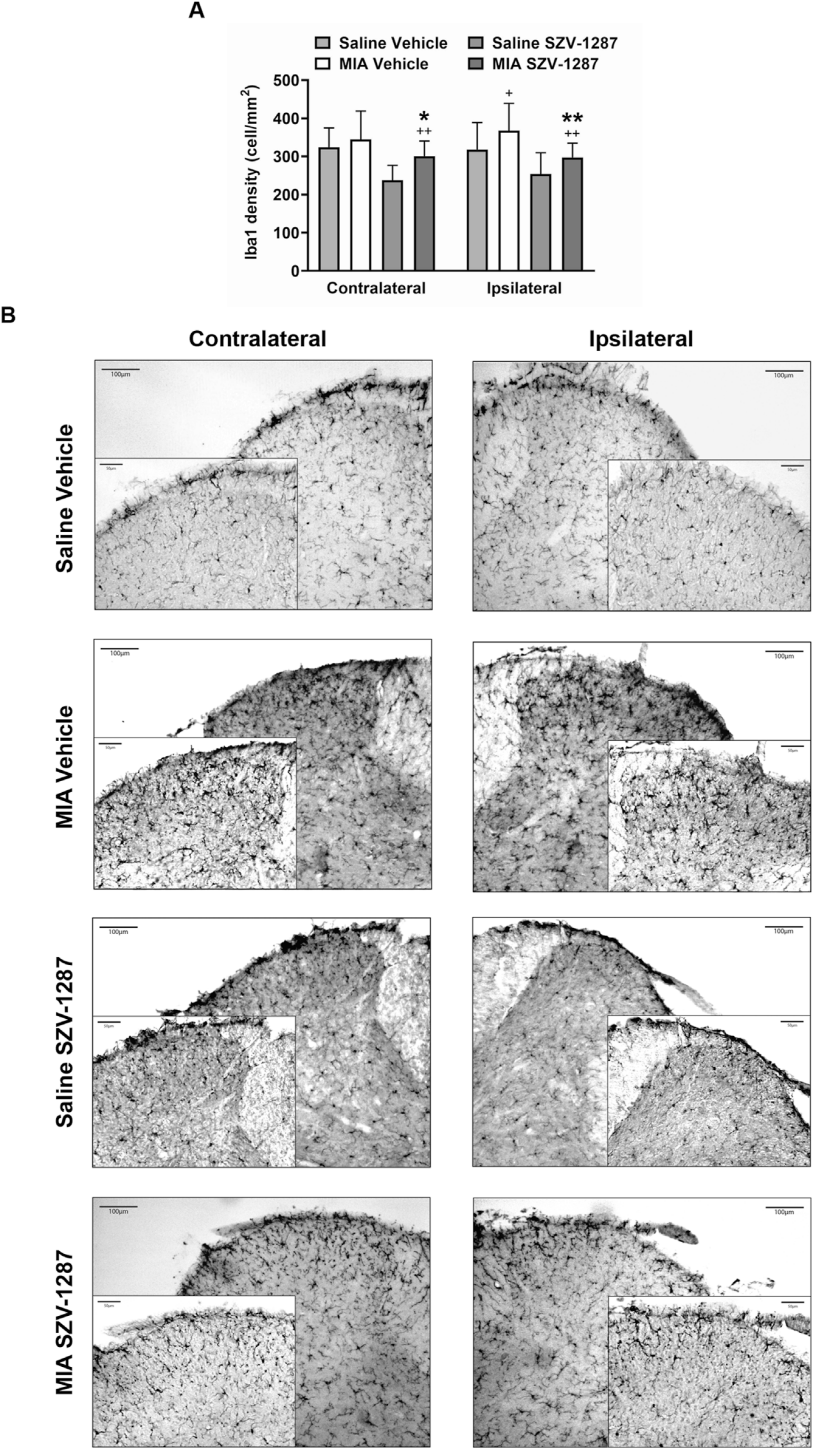
Effect of SZV-1287 on MIA-induced microglia density increase in the lumbar region of the spinal dorsal horn. **(A)** Quantitative analyses and **(B)** representative images of the microglia marker ionized calcium-binding adaptor molecule 1 (Iba1) immunoreactivity (× 10 magnification) in the contralateral and ipsilateral L3–L6 spinal dorsal horn of the SZV-1287-treated (20 mg/kg i.p. every day during the 21-day experimental period) 0.5-mg MIA-injected mice compared to the vehicle-treated group on day 22. The data are shown as means with 95% CI of n = 5–7 mice/group, 2–4 sections/mouse, ^+^g > 0.5, ^++^ *g* > 0.8 vs. respective saline-injected group, ^*^ *g* > 0.5, **g > 0.8 vs. vehicle-treated group. *G* indicates the effect size calculated by Hedges’ g.

The GFAP densities indicating the astrocyte numbers increased in both the contralateral and ipsilateral dorsal horns (Rexed laminae I-II) of the L3–L6 segments of the SC with large effect sizes, while the Iba1 densities demonstrated the microglia numbers in the ipsilateral side with medium effect sizes 22 days after administration of 0.5 mg of MIA compared to the saline-injected group (Figures 7, 8; Supplementary Table S23). Saline injection increased GFAP but not Iba1 density in the ipsilateral spinal dorsal horn with a medium effect size compared to the contralateral side (Figures 7, 8; Supplementary Table S24). SZV-1287 treatment reduced both astrocyte and microglia densities with large effect sizes in the ipsilateral dorsal horn and with medium effect sizes in the contralateral spinal dorsal horn compared to the vehicle-treated group (Figures 7, 8; Supplementary Table S25). No differences were observed between the groups for lateral PAG and SSC-hind limb representations (Supplementary Tables S26–S28).

## 4 Discussion

We show here that our multitarget analgesic candidate SZV-1287 inhibits both spontaneous and referred pain-related behaviors as well as some inflammatory parameters and tissue damage in the MIA-induced mouse OA model, indicating potential disease-modifying effects. The SZV-1287-induced analgesic effects were observed in the late stage of the model, including neuropathic mechanisms, presumably via decreased central nociceptive processing and sensitization. Furthermore, we provide integrative characterization of the MIA model with complex functional, morphological, and *in vivo* imaging analyses related to both inflammatory and nociceptive mechanisms.

Daily i.p. SZV-1287 treatment inhibited MIA-induced late weight bearing deficit, mechanical hyperalgesia, edema, neutrophil MPO activity, and histopathological damage in the affected hind limbs. In addition to the inhibitory effect on pain behaviors, there were parallel decreases in the MIA-induced late astrocyte and microglia number increases in the spinal dorsal horn. This inhibitory action on the neuroinflammatory component might contribute to the reduction of central pain sensitization (Sagar et al., 2011). Several mechanisms can explain the analgesic effects of the compound. The predominant role of AOC3 inhibition and consequently decreased production of TRPA1 activator tissue irritants, such as formaldehyde, methylglyoxal, and hydrogen peroxide, resulting in decreased activation of the primary sensory neurons is suggested. However, direct TRPA1 and TRPV1 antagonism of SZV-1287 that was demonstrated earlier (Payrits et al., 2016) could also contribute to the analgesic effects. The important roles of both receptors in OA pain are well-established (Horvath et al., 2016; Moilanen et al., 2015; Kelly et al., 2015). Moreover, after enzymatic and chemical biotransformation (due to acidic pH of the inflamed area) of SZV-1287, oxaprozin is formed as the main active metabolite, which could also contribute to the therapeutic effects as a non-selective COX-inhibitor that reduces prostaglandin formation. Oxaprozin is a widely used NSAID in the United States that is preferred in the treatment of rheumatic diseases (e.g., RA and OA) owing to its accumulation in the synovial tissues and cartilage as well as ability to increase proteoglycan production (Dallegri et al., 2005). Since prostaglandins are known TRPV1 sensitizers (Ma et al., 2017) and there are several cross-talks between TRPV1 and TRPA1 (Spahn et al., 2014), interactions of SZV-1287 and oxaprozin in the inflamed tissues may also be involved in the overall analgesic effects. Based on its unique multimode action, SZV-1287 may have disease-modifying potential, which meets the current requirements of OA drug development (Spil et al., 2019; Oo et al., 2021; Rodriguez-Merchan, 2023). In addition to its analgesic effect, it exerts anti-inflammatory and chondroprotective actions, which are presumably also related to AOC3 inhibition (Filip et al., 2016), as well as indirect and direct TRPA1 blockade (Moilanen et al., 2015). The important roles of AOC3 and TRPA1 have been demonstrated in several inflammatory and degenerative processes of OA (Filip et al., 2016; Moilanen et al., 2015; Horvath et al., 2016). In the endothelium, AOC3 mediates leukocyte extravasation into the inflamed joints (Marttila-Ichihara et al., 2006), while in the chondrocytes, it accelerates hypertrophic differentiation to initiate cartilage degeneration (Filip et al., 2016). TRPA1 activation in the synoviocytes and chondrocytes can induce cartilage destruction by increasing the inflammatory responses (Moilanen et al., 2015; Yin et al., 2018). Therefore, simultaneous pharmacological blockade of these molecules provides novel but unique mechanisms for analgesic drug development with disease-modifying potential (Marttila-Ichihara et al., 2006; Filip et al., 2016; Moilanen et al., 2015; Yin et al., 2018; Horvath et al., 2018). SZV-1287 may be particularly effective against OA pain because it inhibits both inflammatory and neuropathic components without any hazardous long-term side effects, in contrast to the current first-line NSAID therapy. The drug developmental potential of AOC3/VAP-1 inhibitors in OA is also highlighted by the completed phase II clinical trial on PRX167700 addition to existing NSAID therapy in patients with moderate-to-severe knee OA pain (Alcaraz et al., 2019). In our earlier studies on RA (Horvath et al., 2017) as well as traumatic neuropathy (Horvath et al., 2018) and diabetic neuropathy rodent models (Tékus et al., 2020), the selective AOC3 inhibitor reference compound LJP-1207 exerted similar analgesic and anti-inflammatory effects but induced severe cartilage damage (Horvath et al., 2017).

Broad ranges of MIA doses and experimental paradigms have been used in prior research efforts to model different components of the pathophysiological cascades, which make comparisons and conclusions difficult (Harvey and Dickenson, 2009; Ogbonna et al., 2013; Moilanen et al., 2015; Pitcher et al., 2016; McDougall et al., 2017). Therefore, parallelly with the investigation of the effects of SZV-1287, we characterized the MIA model using two different doses (0.5 and 0.8 mg) with complex investigational techniques related to both inflammatory and nociceptive mechanisms. Despite the severe histopathological damage, mechanical hyperalgesia and weight bearing deficit in the 0.5 mg MIA group decreased gradually until the end of studies supporting the clinical observation that structural abnormalities and pain are not always well-correlated in OA (Spil et al., 2019). In the 0.8 mg MIA group, weight bearing asymmetry was minimally greater in the late phase of the model (on days 15 and 21) compared to the 0.5 mg MIA group, but similar to the mechanical hyperalgesia and histopathological damage, no statistical differences were observed between two MIA doses during the experimental period. When applying similar MIA doses (0.5 and 0.75 mg in mice or 0.5 and 1 mg in rats), dose dependency was not observed with regard to mechanical hyperalgesia in mice (Ogbonna et al., 2013; Pitcher et al., 2016) and histopathological alterations in rats (Udo et al., 2016), while spontaneous weight bearing asymmetry measured through the classic static incapacitance tester only developed after 0.75 and 1 mg MIA administration (Ogbonna et al., 2013; Pitcher et al., 2016). Hind paw mechanical hyperalgesia decreased gradually from week 4 to 6 after 3 mg MIA administration in rats despite the persistent weight bearing asymmetry (Aso et al., 2016), which is in agreement with the findings of our mouse model.

Neutrophil MPO activity as an indicator of cellular inflammatory responses was already elevated 3 h after MIA injection compared to the non-injected knees and increased further on day 1. The enzyme MPO is primarily responsible for neutrophil reactive oxygen species (ROS) production, which also peaks on day 1 in rats (Xie et al., 2012). Activities of the MMPs, including MMP-3 and -13 that are key degrading enzymes in OA pathology (Blom et al., 2007; Baragi et al., 2009), were higher on day 4 compared to the non-injected knees and were gradually relieved by the end of the study. Vascular pathology, particularly synovial and subchondral neovascularization, can also contribute to OA progression (Walsh et al., 2007a). Fluorescence increases in the MIA-injected knees also indicate increased vascular leakage caused by inflammatory vascular responses, such as hyperpermeability and neovascularization, in the initial phase. Increased vascularity in the entire knee and distal femur were also observed in rats 3 weeks after injection of 1 mg of MIA based on contrast-enhanced micro-CT (Xie et al., 2012). All of these data suggest the important roles of ROS production, MMPs, and inflammatory vascular changes in the initiation of OA.

Increased fluorescence signals induced by a bisphosphonate imaging probe were detected, indicating increased bone turnover and showing the correlation between metabolic activity and structural alterations. Bone resorption characterized by volume loss and decreased trabeculation as well as cortical erosions characterized by higher open porosity were observed in the distal femoral and proximal tibial epiphyses. These pilot results from the mouse model are mostly supported by previous studies on rats (Mohan et al., 2011; Xie et al., 2012).

It is well known that central sensitization is responsible for pain persistence in the late stage of the model, which is at least partially mediated by neuroinflammatory processes such as microglia and astrocyte activation (Sagar et al., 2011). Here, bilateral astrogliosis and unilateral microgliosis were observed in the dorsal horn of the lumbar spinal cord 22 days after injection of 0.5 mg of MIA compared to saline, which could explain the dynamic weight bearing asymmetry and referred mechanical hyperalgesia. The higher contralateral astrocyte density could be explained by the greater loading of the contralateral limb due to MIA-induced knee OA, as shown by the histopathology and dynamic weight distribution. GFAP density increase seems to be a very sensitive parameter for mild neuroinflammatory changes. Our result in this regard is partially supported by previous rat studies showing both microgliosis and astrogliosis in the ipsilateral spinal dorsal horn 28 days after MIA injections (Orita et al., 2011; Sagar et al., 2011). However, in mice, spinal microgliosis but not astrogliosis was observed at this timepoint compared to the contralateral side and saline-injected group (Ogbonna et al., 2013).

It is noted that one of the main limitations of this study is the use of only male mice. Although sex difference have been established in the MIA-induced rat (Ro et al., 2020) as well as mouse post-traumatic and collagenase-induced OA (von Loga et al., 2020; Temp et al., 2020; Blaker et al., 2021; Valdrighi et al., 2023) models, our primary aim was to provide a proof-of-concept idea regarding the analgesic and disease-modifying effects of the drug candidate SZV-1287. Exploring sex-dependent changes was beyond the scope of this study but will have relevance in future experiments. The other limitation of this work is the pretreatment paradigm that was used with reduced translational value. However, owing to the robust, very early, and irreversible cartilage degeneration caused by MIA (O’Brien et al., 2017; Bapat et al., 2018), the post-treatment experimental paradigm is likely not capable of reversing the pathophysiological alterations within the 3-week experimental period. This is also supported by data from literature in which post-treatment protocols are used, when only the potential anti-nociceptive effects are investigated after acute administration (Vonsy et al., 2009; Fernihough et al., 2004).

In conclusion, our unique and innovative multitarget analgesic candidate SZV-1287 could become a promising novel therapy for OA with special emphasis on pain while also exerting anti-inflammatory and chondroprotective effects. Exploration of its disease-modifying effects and underlying mechanisms is planned in the future using a surgically induced OA model that causes less robust cartilage degeneration.

## Data Availability

The original contributions presented in the study are included in the article/Supplementary Material; further inquiries can be directed to the corresponding author.

## Supplementary material

SUPPLEMENTARY FIGURE S1 Comparison of the weight bearing asymmetry and mechanical hyperalgesia effects in the 0.5- and 0.8-mg MIA-induced groups. Dynamic weight bearing of the ipsilateral hind limbs of the vehicle- and SZV-1287-treated (20 mg/kg i.p. every day during the 21-day experimental period) saline, 0.5-mg, and 0.8-mg MIA-injected mice **(A)** compared to the baseline (BL) and **(B-F)** contralateral hind limbs. The data are shown as means with 95% CI of n = 7–29 mice/group, ^$^ *g* > 0.5, ^$$^ *g* > 0.8 vs. respective BL, ^##^ *d* > 0.8 vs. contralateral side. Percentage changes in the withdrawal thresholds of the **(G)** ipsilateral and **(H)** contralateral hind paws of the vehicle-treated mice administered saline or 0.5 and 0.8 mg MIA. The data are shown as means with 95% CI of n = 9–29 mice/group, ^++^ *g* > 0.8 vs. saline-injected group. *G* indicates the effect size calculated by Hedges’ g.

SUPPLEMENTARY FIGURE S2 Effects of SZV-1287 on MIA-induced knee edema. Percentage changes in the anteroposterior (AP) and mediolateral (ML) knee diameters of the vehicle- and SZV-1287-treated (20 mg/kg i.p. every day during the 21-day experimental period) mice administered **(A)** 0.5 and **(B)** 0.8 mg MIA, as compared to the respective saline-injected group. The data are shown as means with 95% CI of n = 7–29 mice/group, ^++^ *g* > 0.8 vs. saline-injected group, **g* > 0.5 vs. vehicle-treated group. *G* indicates the effect size calculated by Hedges’ g.

SUPPLEMENTARY FIGURE S3 Effects of SZV-1287 on MIA-induced microarchitectural changes of the proximal tibia. Quantifications of the **(A)** bone volume density, **(B)** trabecular number (Tb.N), **(C)** trabecular separation (Tb.Sp), **(D)** trabecular pattern factor (Tb.Pf), **(E)** volume of open pore space (Po.V(op)), and **(F)** open porosity (Po(op)) in the contralateral and ipsilateral femoral epiphyses of the SZV-1287-treated (20 mg/kg i.p. every day during the 21-day experimental period) 0.5 mg MIA-injected mice compared to the vehicle-treated group on day 22. The data are shown as means with 95% CI of n = 8–10 mice/group, ^#^ *g* > 0.5, ^##^ *g* > 0.8 vs. respective contralateral side, **g* > 0.5 vs. vehicle-treated group. *G* indicates the effect size calculated by Hedges’ g.
