# Correction: Novel multitarget analgesic candidate SZV-1287 demonstrates potential disease-modifying effects in the monoiodoacetate-induced osteoarthritis mouse model

**DOI:** 10.3389/fphar.2025.1646562

**Published:** 2025-07-11

**Authors:** Ádám István Horváth, Kata Bölcskei, Nikolett Szentes, Éva Borbély, Valéria Tékus, Bálint Botz, Kitti Rusznák, Anett Futácsi, Boldizsár Czéh, Péter Mátyus, Zsuzsanna Helyes

**Affiliations:** ^1^ Department of Pharmacology and Pharmacotherapy, Medical School, University of Pécs, Pécs, Hungary; ^2^ National Laboratory for Drug Research and Development, Budapest, Hungary; ^3^ Hungarian Research Network, HUN-REN-PTE Chronic Pain Research Group, Pécs, Hungary; ^4^ Department of Laboratory Diagnostics, Faculty of Health Sciences, University of Pécs, Pécs, Hungary; ^5^ Department of Medical Imaging, Medical School, University of Pécs, Pécs, Hungary; ^6^ Department of Laboratory Medicine, Medical School, University of Pécs, Pécs, Hungary; ^7^ Neurobiology of Stress Research Group, János Szentágothai Research Centre, University of Pécs, Pécs, Hungary; ^8^ National Laboratory of Infectious Animal Diseases, Antimicrobial Resistance, Veterinary Public Health and Food Chain Safety, University of Veterinary Medicine, Budapest, Hungary; ^9^ PharmInVivo Ltd., Pécs, Hungary; ^10^ ALGONIST Biotechnologies GmBH, Vienna, Austria

**Keywords:** osteoarthritic pain, analgesic drug development, semicarbazide-sensitive amine oxidase, amine oxidase copper containing 3, vascular adhesion protein 1, transient receptor potential vanilloid 1, transient receptor potential ankyrin 1

In the **abstract**, there were mistakes in the first sentence of the Results section which was incorrectly written as “MIA induced remarkably decreased thresholds of weight bearing and paw withdrawal, alterations in the tibial and femoral structures (reactive sclerosis, increased trabeculation, and cortical erosions), histopathological damage (disorganized cartilage structure, hypocellularity, decreased matrix staining and tidemark integrity, and increased synovial hyperplasia and osteophyte formation), and changes in the astrocyte and microglia density in the lumbar spinal cord.”.

This has been corrected to read:

“MIA induced remarkable weight bearing and paw withdrawal threshold decrease, alterations in the tibial and femoral structures (decreased trabeculation and cortical erosions), histopathological damage (disorganized cartilage structure, hypocellularity, decreased matrix staining, disrupted tidemark integrity, synovial hyperplasia, and osteophyte formation), and changes in the astrocyte and microglia density in the lumbar spinal cord.”

In the published article, there was a mistake in the **Introduction** section, Paragraph 3. The incorrect sentence read “The nocifensive behavior persists even after resolution of the acute inflammatory phase (Harvey and Dickenson, 2009), and central sensitization develops when consistent with humans (Kelly et al., 2013; McDougall et al., 2017).”.

This has been corrected to read:

“The nocifensive behavior persists even after resolution of the acute inflammatory phase (Harvey and Dickenson, 2009), and consistent with humans, central sensitization develops (Kelly et al., 2013; McDougall et al., 2017).”

In the published article, there was a mistake in the **Materials and methods** section, *Experimental design,* Paragraph 2. The incorrect sentence read “All measurement timepoints for these complex protocols were chosen on the basis of our experience and data available in literature on the different pathophysiological parameters of the model (Blom et al., 2007; Baragi et al., 2009; Walsh et al., 2007a; Xie et al., 2012).”

This has been corrected to read:

“All measurement timepoints for these complex protocols were chosen on the basis of our experience and literature data available on the different pathophysiological parameters of the model (Blom et al., 2007; Baragi et al., 2009; Walsh et al., 2007a; Xie et al., 2012).”

In the published article, there was a mistake in the **Results** section, *SZV-1287 inhibits MIA-induced histopathological damage to the knee*. The title of the subsection was incorrect and it now has been corrected to read:

“SZV-1287 inhibits MIA-induced histopathological damage of the knee”.

In the published article, there was a mistake in the **Results** section, *SZV-1287 does not alter MIA-induced microarchitectural changes of the bones*, Paragraph 1. The incorrect sentence read “The most prominent MIA-induced alterations of the subchondral trabecular bone microarchitecture were bone resorption (indicated by decreased volume density and trabecular number), increased trabecular separation and pattern factor, and cortical erosions (indicated by increased open pore space volume and open porosity compared to the contralateral side) (Figures 6A–F. Supplementary Figure S3A).”

This has been corrected to read:

“The most prominent MIA-induced alterations of the subchondral trabecular bone microarchitecture were bone resorption (indicated by decreased volume density and trabecular number, as well as increased trabecular separation and pattern factor), and cortical erosions (indicated by increased open pore space volume and open porosity compared to the contralateral side) (Figures 6A–F. Supplementary Figure S3A).”

In the published article, there was a mistake in the **Discussion** section, Paragraph 2**.** The incorrect sentence read “Endothelial AOC3 mediates leukocyte extravasation into the inflamed joints (Marttila-Ichihara et al., 2006) while accelerating hypertrophic differentiation to initiate cartilage degeneration in the chondrocytes (Filip et al., 2016).”.

This has been corrected to read:

“In the endothelium, AOC3 mediates leukocyte extravasation into the inflamed joints (Marttila-Ichihara et al., 2006), while in the chondrocytes, it accelerates hypertrophic differentiation to initiate cartilage degeneration (Filip et al., 2016).”

In the published article, there was a mistake in the **Discussion** section, Paragraph 8. The incorrect sentence read “Exploration of its disease-modifying effects and mechanisms is planned in the future in surgically induced OA models to induce less robust cartilage degeneration.”

This has been corrected to read:

“Exploration of its disease-modifying effects and underlying mechanisms is planned in the future using a surgically induced OA model that causes less robust cartilage degeneration.”

The original version of this article has been updated.

