# Supplementary material for: Novel multitarget analgesic candidate SZV-1287 demonstrates potential disease-modifying effects in the monoiodoacetate-induced osteoarthritis mouse model
Source: Front Pharmacol. 2024 Sep 16;15:1377081. doi: 10.3389/fphar.2024.1377081 (PMC11439770; doi:10.3389/fphar.2024.1377081)
Supplement: Supplementary file 1 [file Table1.docx]

**Supplementary table 1. Comparisons of factors time (baseline vs. experimental days) and drug (vehicle vs. SZV-1287).** The table shows the statistics for weight bearing deficit, mechanical hyperalgesia, and anteroposterior (AP) and mediolateral (ML) knee diameter increase. The statistics were performed using the means with 95% CI of n=7-29 mice/group. *P*<0.05 (mixed-effects model followed by Dunnett’s, Sidak’s or Tukey’s multiple comparisons test in the case of weight bearing deficit, and AP and ML knee diameter increase, two-way repeated measures ANOVA followed by Sidak’s multiple comparisons test in the case of mechanical hyperalgesia) was considered significant indicated by *.

| **Figure panel** | **Variable** | **Baseline vs. Experimental days** | | **Vehicle vs. SZV-1287** | | **Interaction** | |
| --- | --- | --- | --- | --- | --- | --- | --- |
|  |  | **F** | ***p* value** | **F** | ***p* value** | **F** | ***p* value** |
| **Figure 2A** | **Weight bearing deficit** | F (3.033, 113.7) = 29.98 | **<0.0001****** | F (1, 54) = 1.264 | 0.2659 | F (4, 150) = 1.023 | 0.3975 |
| **Figure 2B** | **Weight bearing deficit** | F (3.284, 45.98) = 10.38 | **<0.0001****** | F (1, 14) = 2.325 | 0.1495 | F (4, 56) = 0.7035 | 0.5928 |
| **Figure 2C** | **Mechanical hyperalgesia** | F (3.540, 191.2) = 65.61 | **<0.0001****** | F (1, 54) = 8.217 | **0.0059**** | F (4, 216) = 2.669 | **0.0332*** |
| **Figure 2D** | **Mechanical hyperalgesia** | F (3.048, 42.68) = 47.14 | **<0.0001****** | F (1, 14) = 2.121 | 0.1673 | F (4, 56) = 3.421 | **0.0143*** |
| **Suppl. Figure 2A** | **AP knee diameter** | F (3.705, 188.2) = 102.1 | **<0.0001****** | F (1, 56) = 0.2853 | 0.5953 | F (5, 254) = 0.5518 | 0.7369 |
| **Suppl. Figure 2A** | **ML knee diameter** | F (3.047, 164.6) = 127.1 | **<0.0001****** | F (1, 60) = 0.3829 | 0.5384 | F (5, 270) = 0.4108 | 0.8411 |
| **Suppl. Figure 2B** | **AP knee diameter** | F (3.328, 46.59) = 25.18 | **<0.0001****** | F (1, 14) = 0.6837 | 0.4222 | F (5, 70) = 0.8041 | 0.5506 |
| **Suppl. Figure 2B** | **ML knee diameter** | F (2.895, 40.53) = 42.60 | **<0.0001****** | F (1, 14) = 1.652 | 0.2196 | F (5, 70) = 0.5575 | 0.7321 |

**Supplementary table 2. Comparisons of factors model (saline vs. MIA or contralateral vs. ipsilateral) and drug (vehicle vs. SZV-1287).** The table shows the statistics for neutrophil MPO and MMP activities, vascular leakage, bone remodeling, bone microarchitectural alterations (bone volume density, trabecular number (Tb.N), separation (Tb.Sp) and pattern factor (Tb.Pf), volume of open pore space (Po.V(op)) and open porosity (Po(op)), GFAP and Iba1 densities. The statistics were performed using the means with 95% CI of n=6-12 mice/group in the case of neutrophil MPO activity, n=5-6 mice/group in the case of MMP activity and vascular leakage, n=3-8/group in the case of bone remodeling, n=8-10 mice/group in the case of bone microarchitectural alterations, n=5-8 mice/group in the case of GFAP density and n=5-7 mice /group in the case of Iba1 density. *P*<0.05 (two-way ANOVA followed by Sidak’s multiple comparisons test) was considered significant indicated by *.

| **Figure panel** | **Variable** | **Saline vs. MIA or Contra- vs. Ipsilateral** | | **Vehicle vs. SZV-1287** | | **Interaction** | |
| --- | --- | --- | --- | --- | --- | --- | --- |
|  |  | **F** | ***p* value** | **F** | ***p* value** | **F** | ***p* value** |
| **Figure 4A** | **Neutrophil MPO activity (3 h)** | F (1, 20) = 18.72 | **0.0003**** | F (1, 20) = 6.509 | **0.0190*** | F (1, 20) = 1.777 | 0.1976 |
| **Figure 4A** | **Neutrophil MPO activity (24 h)** | F (1, 42) = 74.94 | **<0.0001****** | F (1, 42) = 19.39 | **<0.0001****** | F (1, 42) = 10.87 | **0.0020**** |
| **Figure 4B** | **MMP activity (Day 4)** | F (1, 18) = 63.97 | **<0.0001****** | F (1, 18) = 0.03100 | 0.8622 | F (1, 18) = 0.1137 | 0.7398 |
| **Figure 4B** | **MMP activity (Day 8)** | F (1, 18) = 11.18 | **0.0036**** | F (1, 18) = 0.004628 | 0.9465 | F (1, 18) = 0.9162 | 0.3511 |
| **Figure 5A** | **Vascular leakage** | F (1, 18) = 53.83 | **<0.0001****** | F (1, 18) = 0.7306 | 0.4039 | F (1, 18) = 0.004344 | 0.9482 |
| **Figure 5C** | **Bone remodeling** | F (1, 18) = 20.51 | **0.0003***** | F (1, 18) = 0.08885 | 0.7691 | F (1, 18) = 1.879 | 0.1873 |
| **Figure 6A** | **Bone volume density** | F (1, 32) = 11.37 | **0.0020**** | F (1, 32) = 5.975 | **0.0202*** | F (1, 32) = 0.7355 | 0.3975 |
| **Figure 6B** | **Tb.N** | F (1, 32) = 8.600 | **0.0062**** | F (1, 32) = 0.04237 | 0.8382 | F (1, 32) = 0.04978 | 0.8249 |
| **Figure 6C** | **Tr.Sp** | F (1, 32) = 3.960 | 0.0552 | F (1, 32) = 0.03501 | 0.8527 | F (1, 32) = 0.4515 | 0.5064 |
| **Figure 6D** | **Tb.Pf** | F (1, 32) = 45.55 | **<0.0001****** | F (1, 32) = 1.369 | 0.2506 | F (1, 32) = 0.006776 | 0.9349 |
| **Figure 6E** | **Po.V(op)** | F (1, 32) = 8.002 | **0.0080**** | F (1, 32) = 1.904 | 0.1772 | F (1, 32) = 0.7777 | 0.3844 |
| **Figure 6F** | **Po(op)** | F (1, 32) = 11.38 | **0.0020**** | F (1, 32) = 5.990 | **0.0201*** | F (1, 32) = 0.7570 | 0.3908 |
| **Figure 7A** | **GFAP density (ipsilateral)** | F (1, 23) = 2.974 | 0.0980 | F (1, 23) = 1.307 | 0.2648 | F (1, 23) = 2.464 | 0.1301 |
| **Figure 7A** | **GFAP density (contra-lateral)** | F (1, 23) = 5.256 | 0.0313 | F (1, 23) = 0.3598 | 0.5545 | F (1, 23) = 2.773 | 0.1095 |
| **Figure 8A** | **Iba1 density (ipsilateral)** | F (1, 21) = 4.842 | **0.0391*** | F (1, 21) = 9.476 | **0.0057**** | F (1, 21) = 0.0009613 | 0.9756 |
| **Figure 8A** | **Iba1 density (contra-lateral)** | F (1, 21) = 4.652 | **0.0427*** | F (1, 21) = 11.17 | **0.0031**** | F (1, 21) = 1.192 | 0.2873 |
| **Suppl. Figure 3A** | **Bone volume density** | F (1, 32) = 3.528 | 0.0695 | F (1, 32) = 4.896 | **0.0342*** | F (1, 32) = 0.01933 | 0.8903 |
| **Suppl. Figure 3B** | **Tb.N** | F (1, 32) = 6.598 | **0.0151*** | F (1, 32) = 0.3726 | 0.5459 | F (1, 32) = 0.3678 | 0.5485 |
| **Suppl. Figure 3C** | **Tr.Sp** | F (1, 32) = 1.913 | 0.1762 | F (1, 32) = 1.577 | 0.2184 | F (1, 32) = 0.2407 | 0.6270 |
| **Suppl. Figure 3D** | **Tb.Pf** | F (1, 32) = 7.064 | **0.0122*** | F (1, 32) = 1.504 | 0.2291 | F (1, 32) = 0.01219 | 0.9128 |
| **Suppl. Figure 3E** | **Po.V(op)** | F (1, 32) = 2.557 | 0.1196 | F (1, 32) = 0.3504 | 0.5581 | F (1, 32) = 0.07243 | 0.7896 |
| **Suppl. Figure 3F** | **Po(op)** | F (1, 32) = 3.449 | 0.0725 | F (1, 32) = 4.706 | **0.0376*** | F (1, 32) = 0.04619 | 0.8312 |

**Supplementary table 3. Comparisons of factors time (baseline vs. experimental days) and model (saline vs. MIA or contralateral vs. ipsilateral).** The table shows the statistics for weight bearing deficit, mechanical hyperalgesia, and anteroposterior (AP) and mediolateral (ML) knee diameter increase. The statistics were performed using the means with 95% CI of n=7-29 mice/group. *P*<0.05 (mixed-effects model followed by Dunnett’s, Sidak’s or Tukey’s multiple comparisons test in the case of weight bearing deficit, and AP and ML knee diameter increase, two-way repeated measures ANOVA followed by Sidak’s multiple comparisons test in the case of mechanical hyperalgesia) was considered significant indicated by *.

| **Figure panel** | **Variable** | **Baseline vs. Experimental days** | | **Saline vs. MIA or**  **Contra- vs. Ipsilateral** | | **Interaction** | |
| --- | --- | --- | --- | --- | --- | --- | --- |
|  |  | **F** | ***p* value** | **F** | ***p* value** | **F** | ***p* value** |
| **Suppl. Figure 1A** | **Weight bearing deficit** | F (3.136, 104.3) = 16.69 | **<0.0001****** | F (2, 48) = 2.104 | 0.1331 | F (8, 133) = 2.157 | **0.0347*** |
| **Suppl. Figure 1B** | **Weight bearing deficit** | F (2.466, 30.83) = 1.529e-014 | >0.9999 | F (1, 24) = 37.76 | **<0.0001****** | F (4, 50) = 7.066 | **0.0001****** |
| **Suppl. Figure 1C** | **Weight bearing deficit** | F (3.106, 118.0) = 3.701e-008 | >0.9999 | F (1, 56) = 98.89 | **<0.0001****** | F (4, 152) = 34.57 | **<0.0001****** |
| **Suppl. Figure 1D** | **Weight bearing deficit** | F (2.637, 42.19) = 5.802e-029 | >0.9999 | F (1, 16) = 54.41 | **<0.0001****** | F (4, 64) = 14.63 | **<0.0001****** |
| **Suppl. Figure 1E** | **Weight bearing deficit** | F (2.371, 87.71) = 1.234e-008 | >0.9999 | F (1, 52) = 63.91 | **<0.0001****** | F (4, 148) = 28.88 | **<0.0001****** |
| **Suppl. Figure 1F** | **Weight bearing deficit** | F (2,500, 30.00) = 4.521e-029 | >0.9999 | F (1, 12) = 20.58 | **0.0007***** | F (4, 48) = 8.322 | **<0.0001****** |
| **Suppl. Figure 1G** | **Mechanical hyperalgesia** | F (3.446, 165.4) = 38.65 | **<0.0001****** | F (2, 48) = 27.23 | **<0.0001****** | F (8, 192) = 6.223 | **<0.0001****** |
| **Suppl. Figure 1H** | **Mechanical hyperalgesia** | F (3.268, 156.9) = 0.8803 | 0.4601 | F (2, 48) = 0.02794 | 0.9725 | F (8, 192) = 1.379 | 0.2079 |
| **Suppl. Figure 2A** | **AP knee diameter** | F (3.684, 118.6) = 15.78 | **<0.0001****** | F (1, 34) = 68.22 | **<0.0001****** | F (5, 161) = 14.22 | **<0.0001****** |
| **Suppl. Figure 2A** | **ML knee diameter** | F (3.347, 107.8) = 14.44 | **<0.0001****** | F (1, 34) = 32.50 | **<0.0001****** | F (5, 161) = 13.70 | **<0.0001****** |
| **Suppl. Figure 2B** | **AP knee diameter** | F (3.243, 45.40) = 10.72 | **<0.0001****** | F (1, 14) = 38.66 | **<0.0001****** | F (5, 70) = 10.47 | **<0.0001****** |
| **Suppl. Figure 2B** | **ML knee diameter** | F (3.270, 45.79) = 15.01 | **<0.0001****** | F (1, 14) = 31.20 | **<0.0001****** | F (5, 70) = 14.79 | **<0.0001****** |

**Supplementary table 4. Comparison of the dynamic weight bearing of the ipsilateral hind limbs with the baseline.** The table shows the statistics for dynamic weight bearing of the ipsilateral hind limbs of vehicle- and SZV-1287-treated (20 mg/kg i.p. every day during the 21-day experimental period) saline-, 0.5 mg and 0.8 mg monoiodoacetate (MIA)-injected mice as compared to the baseline. The statistics were performed using the means with 95% CI of n=7-29 mice/group. *P*<0.05 (mixed-effects model followed by Dunnett’s multiple comparisons test) was considered significant indicated by * and effect size was calculated using Hedges’ *g*.

| **Groups** | **In comparison with the baseline** | | | |
| --- | --- | --- | --- | --- |
|  | **Day 2** | **Day 9** | **Day 15** | **Day 21** |
|  |  |  |  |  |
| **Saline Vehicle** |  |  |  |  |
| *p* value | 0.7668 | 0.7058 | 0.9294 | 0.1205 |
| Hedges’ *g* (effect size) | **0.7 ↓** | **0.6 ↓** | 0.3 | **0.9 ↑** |
|  |  |  |  |  |
| **0.5 mg MIA Vehicle** |  |  |  |  |
| *p* value | **<0.0001****** | **0.0002***** | **0.0039**** | **0.009**** |
| Hedges’ *g* (effect size) | **2.1 ↓** | **1.9 ↓** | **1.2 ↓** | **0.8 ↓** |
|  |  |  |  |  |
| **0.5 mg MIA SZV-1287** |  |  |  |  |
| *p* value | **0.0007***** | **<0.0001****** | 0.0533 | **0.0039**** |
| Hedges’ *g* (effect size) | **1.7 ↓** | **3.1 ↓** | **0.9 ↓** | **0.9 ↓** |
|  |  |  |  |  |
| **0.8 mg MIA Vehicle** |  |  |  |  |
| *p* value | **0.0119*** | **0.0026**** | **0.0296*** | **0.0255*** |
| Hedges’ *g* (effect size) | **1.7 ↓** | **2.2 ↓** | **1.3 ↓** | **1.4↓** |
|  |  |  |  |  |
| **0.8 mg MIA SZV-1287** |  |  |  |  |
| *p* value | 0.1065 | 0.1074 | 0.9678 | 0.8101 |
| Hedges’ *g* (effect size) | **1.5 ↓** | **1.5 ↓** | 0.2 | 0.4 |

**Supplementary table 5. Comparison of the dynamic weight bearing of the ipsilateral hind limbs with the contralateral side.** The table shows the statistics for dynamic weight bearing of the ipsilateral hind limbs of vehicle- and SZV-1287-treated (20 mg/kg i.p. every day during the 21-day experimental period) saline-, 0.5 mg and 0.8 mg monoiodoacetate (MIA)-injected mice as compared to the respective contralateral side. The statistics were performed using the means with 95% CI of n=7-29 mice/group. *P*<0.05 (mixed-effects model followed by Sidak’s multiple comparisons test for weight bearing of saline- and 0.5 mg MIA-injected mice, two-way repeated measures ANOVA followed by Sidak’s multiple comparisons test for weight bearing of 0.8 mg MIA-injected mice) was considered significant indicated by * and effect size was calculated using Hedges’ *g*.

| **Groups** | **In comparison with the contralateral side** | | | |
| --- | --- | --- | --- | --- |
|  | **Day 2** | **Day 9** | **Day 15** | **Day 21** |
|  |  |  |  |  |
| **Saline Vehicle** |  |  |  |  |
| *p* value | **0.0181*** | **0.0379*** | **0.006**** | 0.0806 |
| Hedges’ *g* (effect size) | **1.3 ↓** | **2.2 ↓** | **2.6 ↓** | **1.7 ↓** |
|  |  |  |  |  |
| **0.5 mg MIA Vehicle** |  |  |  |  |
| *p* value | 0.7972 | **<0.0001****** | **<0.0001****** | **0.0005***** |
| Hedges’ *g* (effect size) | 0.3 | **2.8 ↓** | **2.4 ↓** | **1.5 ↓** |
|  |  |  |  |  |
| **0.5 mg MIA SZV-1287** |  |  |  |  |
| *p* value | 0.3840 | **<0.0001****** | **<0.0001****** | **0.0343*** |
| Hedges’ *g* (effect size) | 0.5 | **2.1 ↓** | **4.2 ↓** | **1 ↓** |
|  |  |  |  |  |
| **0.8 mg MIA Vehicle** |  |  |  |  |
| *p* value | 0.5598 | **0.0001***** | **<0.0001****** | **0.001***** |
| Hedges’ *g* (effect size) | **0.7 ↑** | **2.7 ↓** | **3.6 ↓** | **2.3 ↓** |
|  |  |  |  |  |
| **0.8 mg MIA SZV-1287** |  |  |  |  |
| *p* value | 0.7607 | **0.0014**** | **0.0022**** | 0.7920 |
| Hedges’ *g* (effect size) | **0.7 ↑** | **2.7 ↓** | **2.6 ↓** | **0.6 ↓** |

**Supplementary table 6. Comparison of the dynamic weight bearing of the ipsilateral hind limbs of vehicle- and SZV-1287-treated mice.** The table shows the statistics for dynamic weight bearing of the ipsilateral hind limbs of SZV-1287-treated (20 mg/kg i.p. every day during the 21-day experimental period) 0.5 mg and 0.8 mg monoiodoacetate (MIA)-injected mice as compared to the respective vehicle-treated group. The statistics were performed using the means with 95% CI of n=7-29 mice/group. *P*<0.05 (mixed-effects model followed by Sidak’s multiple comparisons test in case of 0.5 mg MIA-injected mice, two-way repeated measures ANOVA followed by Sidak’s multiple comparisons test in case of 0.8 mg MIA-injected mice) was considered significant indicated by * and effect size was calculated using Hedges’ *g*.

| **Groups** | **In comparison with the vehicle-treated group** | | | | |
| --- | --- | --- | --- | --- | --- |
|  | **Baseline** | **Day 2** | **Day 9** | **Day 15** | **Day 21** |
|  |  |  |  |  |  |
| **0.5 mg MIA** |  |  |  |  |  |
| *p* value | 0.9985 | 0.6957 | 0.4813 | >0.9999 | 0.8729 |
| Hedges’ *g* (effect size) | 0.1 | 0.4 | 0.5 | 0.1 | 0.3 |
|  |  |  |  |  |  |
| **0.8 mg MIA** |  |  |  |  |  |
| *p* value | >0.9999 | 0.8488 | 0.9389 | 0.4824 | 0.5290 |
| Hedges’ *g* (effect size) | 0.1 | 0.5 | 0.4 | **0.8 ↑** | **0.8 ↑** |

**Supplementary table 7. Comparison of the mechanical hyperalgesia of the ipsilateral hind paws.** The table shows the statistics for mechanical hyperalgesia of the ipsilateral hind paws of vehicle- and SZV-1287-treated (20 mg/kg i.p. every day during the 21-day experimental period) saline-, 0.5 mg and 0.8 mg monoiodoacetate (MIA)-injected mice. The statistics were performed using the means with 95% CI of n=7-29 mice/group. *P*<0.05 (two-way repeated measures ANOVA followed by Tukey’s multiple comparisons test) was considered significant indicated by * and effect size was calculated using Hedges’ *g*.

| **Group comparisons** | **Day 2** | **Day 9** | **Day 15** | **Day 21** |
| --- | --- | --- | --- | --- |
|  |  |  |  |  |
| **Saline vs. 0.5 mg MIA Vehicle** |  |  |  |  |
| *p* value | **<0.0001****** | **0.0009***** | **0.0017**** | **0.0004***** |
| Hedges’ *g* (effect size) | **2.3 ↓** | **1.1 ↓** | **1.5 ↓** | **1.2 ↓** |
|  |  |  |  |  |
| **Saline vs. 0.8 mg MIA Vehicle** |  |  |  |  |
| *p* value | **<0.0001****** | **<0.0001****** | **0.0009***** | **0.0004***** |
| Hedges’ *g* (effect size) | **3.6 ↓** | **2.2 ↓** | **1.7 ↓** | **2.1 ↓** |
|  |  |  |  |  |
| **0.5 mg vs. 0.8 mg MIA Vehicle** |  |  |  |  |
| *p* value | 0.6554 | 0.4956 | 0.8242 | 0.6037 |
| Hedges’ *g* (effect size) | 0.3 | 0.3 | 0.2 | 0.3 |
|  |  |  |  |  |
| **0.5 mg MIA Vehicle vs. SZV-1287** |  |  |  |  |
| *p* value | 0.4263 | 0.2730 | 0.0634 | **0.0068**** |
| Hedges’ *g* (effect size) | 0.4 | 0.5 | **0.7 ↓** | **0.9 ↓** |
|  |  |  |  |  |
| **0.8 mg MIA Vehicle vs. SZV-1287** |  |  |  |  |
| *p* value | 0.9992 | 0.1411 | 0.3857 | 0.9795 |
| Hedges’ *g* (effect size) | 0.1 | **1.3 ↓** | **0.9 ↓** | 0.2 |

**Supplementary table 8. Withdrawal thresholds of the ipsilateral hind paws of vehicle-treated mice.** The table shows the means of withdrawal thresholds of the ipsilateral hind paws of vehicle-treated saline-, 0.5 mg and 0.8 mg monoiodoacetate (MIA)-injected mice with 95% CI of n=7-29 mice/group.

| Time | **Saline** | | | **0.5 mg MIA** | | | **0.8 mg MIA** | | |
| --- | --- | --- | --- | --- | --- | --- | --- | --- | --- |
|  | Mean (g) | Upper limit | Lower limit | Mean (g) | Upper limit | Lower limit | Mean (g) | Upper limit | Lower limit |
|  |  |  |  |  |  |  |  |  |  |
| **Baseline** | 7.72 | 8.09 | 7.36 | 8.30 | 8.53 | 8.08 | 7.82 | 8.32 | 7.32 |
|  |  |  |  |  |  |  |  |  |  |
| **Day 2** | 7.25 | 7.63 | 6.88 | 5.84 | 6.35 | 5.33 | 5.24 | 5.86 | 4.61 |
|  |  |  |  |  |  |  |  |  |  |
| **Day 9** | 7.46 | 8.10 | 6.83 | 6.09 | 6.52 | 5.66 | 5.47 | 5.93 | 5.00 |
|  |  |  |  |  |  |  |  |  |  |
| **Day 15** | 7.57 | 8.36 | 6.79 | 6.45 | 6.76 | 6.14 | 6.00 | 6.20 | 5.80 |
|  |  |  |  |  |  |  |  |  |  |
| **Day 21** | 7.73 | 8.24 | 7.23 | 6.81 | 7.22 | 6.41 | 6.18 | 6.70 | 5.65 |

**Supplementary table 9. Withdrawal thresholds of the contralateral hind paws of vehicle-treated mice.** The table shows the means of withdrawal thresholds of the contralateral hind paws of vehicle-treated saline-, 0.5 mg and 0.8 mg monoiodoacetate (MIA)-injected mice with 95% CI of n=7-29 mice/group.

| Time | **Saline** | | | **0.5 mg MIA** | | | **0.8 mg MIA** | | |
| --- | --- | --- | --- | --- | --- | --- | --- | --- | --- |
|  | Mean (g) | Upper limit | Lower limit | Mean (g) | Upper limit | Lower limit | Mean (g) | Upper limit | Lower limit |
|  |  |  |  |  |  |  |  |  |  |
| **Baseline** | 7.81 | 8.58 | 7.04 | 8.38 | 8.60 | 8.16 | 8.26 | 8.63 | 7.89 |
|  |  |  |  |  |  |  |  |  |  |
| **Day 2** | 7.03 | 7.55 | 6.51 | 8.04 | 8.46 | 7.62 | 8.05 | 8.56 | 7.53 |
|  |  |  |  |  |  |  |  |  |  |
| **Day 9** | 7.24 | 7.66 | 6.83 | 8.22 | 8.51 | 7.93 | 8.11 | 8.51 | 7.72 |
|  |  |  |  |  |  |  |  |  |  |
| **Day 15** | 7.43 | 8.12 | 6.74 | 8.13 | 8.43 | 7.84 | 7.82 | 8.39 | 7.24 |
|  |  |  |  |  |  |  |  |  |  |
| **Day 21** | 7.78 | 8.56 | 7.00 | 8.33 | 8.64 | 8.01 | 8.10 | 8.78 | 7.42 |

**Supplementary table 10. Withdrawal thresholds of the ipsilateral hind paws SZV-1287-treated mice.** The table shows the means of withdrawal thresholds of the ipsilateral hind paws of SZV-1287-treated (20 mg/kg i.p. every day during the 21-day experimental period) saline-, 0.5 mg and 0.8 mg monoiodoacetate (MIA)-injected mice with 95% CI of n=7-29 mice/group.

| Time | **Saline** | | | **0.5 mg MIA** | | | **0.8 mg MIA** | | |
| --- | --- | --- | --- | --- | --- | --- | --- | --- | --- |
|  | Mean (g) | Upper limit | Lower limit | Mean (g) | Upper limit | Lower limit | Mean (g) | Upper limit | Lower limit |
|  |  |  |  |  |  |  |  |  |  |
| **Baseline** | 7.73 | 8.04 | 7.42 | 8.38 | 8.58 | 8.18 | 8.02 | 8.46 | 7.57 |
|  |  |  |  |  |  |  |  |  |  |
| **Day 2** | 7.53 | 8.37 | 6.68 | 6.37 | 6.86 | 5.88 | 5.40 | 6.14 | 4.66 |
|  |  |  |  |  |  |  |  |  |  |
| **Day 9** | 7.23 | 7.95 | 6.51 | 6.89 | 7.41 | 6.36 | 6.68 | 7.49 | 5.88 |
|  |  |  |  |  |  |  |  |  |  |
| **Day 15** | 7.47 | 7.99 | 6.95 | 7.25 | 7.69 | 6.82 | 6.77 | 7.48 | 6.06 |
|  |  |  |  |  |  |  |  |  |  |
| **Day 21** | 7.28 | 7.60 | 6.97 | 7.86 | 8.30 | 7.42 | 6.22 | 6.86 | 5.59 |

**Supplementary table 11. Comparison of the change of anteroposterior diameter of the ipsilateral knees.** The table shows the statistics for change of anteroposterior diameter of the ipsilateral knees of vehicle- and SZV-1287-treated (20 mg/kg i.p. every day during the 21-day experimental period) saline-, 0.5 mg and 0.8 mg monoiodoacetate (MIA)-injected mice. The statistics were performed using the means with 95% CI of n=7-29 mice/group. *P*<0.05 (mixed-effects model followed by Tukey’s multiple comparisons test) was considered significant indicated by * and effect size was calculated using Hedges’ *g*.

| **Group comparisons** | **Hour 3** | **Hour 6** | **Day 1** | **Day 2** | **Day 9** |
| --- | --- | --- | --- | --- | --- |
|  |  |  |  |  |  |
| **Saline vs. 0.5 mg MIA Vehicle** |  |  |  |  |  |
| *p* value | **0.0075**** | **<0.0001****** | **0.0004***** | **0.0008***** | **0.001***** |
| Hedges’ *g* (effect size) | **2.4 ↑** | **2.9 ↑** | **2.8 ↑** | **1.9 ↑** | **1.3** **↑** |
|  |  |  |  |  |  |
| **Saline vs. 0.8 mg MIA Vehicle** |  |  |  |  |  |
| *p* value | **0.002**** | **<0.0001****** | **0.0058**** | **0.0002***** | **0.0026**** |
| Hedges’ *g* (effect size) | **2.2 ↑** | **3.3 ↑** | **1.8 ↑** | **2.8 ↑** | **2 ↑** |
|  |  |  |  |  |  |
| **0.5 mg vs. 0.8 mg MIA Vehicle** |  |  |  |  |  |
| *p* value | 0.3701 | 0.7273 | 0.8135 | 0.3046 | 0.5089 |
| Hedges’ *g* (effect size) | **0.7 ↑** | 0.3 | 0.3 | **0.6 ↑** | 0.4 |
|  |  |  |  |  |  |
| **0.5 mg MIA Vehicle vs. SZV-1287** |  |  |  |  |  |
| *p* value | 0.9814 | 0.8391 | 0.9237 | 0.8659 | >0.9999 |
| Hedges’ *g* (effect size) | 0.1 | 0.2 | 0.2 | 0.2 | 0 |
|  |  |  |  |  |  |
| **0.8 mg MIA Vehicle vs. SZV-1287** |  |  |  |  |  |
| *p* value | 0.9116 | 0.6115 | 0.8387 | 0.9807 | 0.9782 |
| Hedges’ *g* (effect size) | 0.3 | **0.6 ↓** | 0.4 | 0.2 | 0.2 |

**Supplementary table 12. Comparison of the change of mediolateral diameter of the ipsilateral knees.** The table shows the statistics for change of mediolateral diameter of the ipsilateral knees of vehicle- and SZV-1287-treated (20 mg/kg i.p. every day during the 21-day experimental period) saline-, 0.5 mg and 0.8 mg monoiodoacetate (MIA)-injected mice. The statistics were performed using the means with 95% CI of n=7-29 mice/group. *P*<0.05 (mixed-effects model followed by Tukey’s multiple comparisons test) was considered significant indicated by * and effect size was calculated using Hedges’ *g*.

| **Group comparisons** | **Hour 3** | **Hour 6** | **Day 1** | **Day 2** | **Day 9** |
| --- | --- | --- | --- | --- | --- |
|  |  |  |  |  |  |
| **Saline vs. 0.5 mg MIA Vehicle** |  |  |  |  |  |
| *p* value | **0.0003***** | **<0.0001****** | **<0.0001****** | **0.0057**** | 0.2086 |
| Hedges’ *g* (effect size) | **2.5 ↑** | **2.2 ↑** | **2.1 ↑** | **1.8 ↑** | **0.8 ↑** |
|  |  |  |  |  |  |
| **Saline vs. 0.8 mg MIA Vehicle** |  |  |  |  |  |
| *p* value | **<0.0001****** | **0.0001***** | **0.0012**** | **0.0002***** | **0.048*** |
| Hedges’ *g* (effect size) | **2.9 ↑** | **3 ↑** | **2.3 ↑** | **2.7 ↑** | **1.3 ↑** |
|  |  |  |  |  |  |
| **0.5 mg vs. 0.8 mg MIA Vehicle** |  |  |  |  |  |
| *p* value | 0.3371 | 0.9183 | 0.9947 | **0.0328*** | 0.3506 |
| Hedges’ *g* (effect size) | **0.6 ↑** | 0.1 | 0 | **1.4 ↑** | **0.6 ↑** |
|  |  |  |  |  |  |
| **0.5 mg MIA Vehicle vs. SZV-1287** |  |  |  |  |  |
| *p* value | 0.7998 | 0.7905 | 0.9996 | 0.9651 | 0.8909 |
| Hedges’ *g* (effect size) | 0.3 | 0.4 | 0.1 | 0.2 | 0.5 |
|  |  |  |  |  |  |
| **0.8 mg MIA Vehicle vs. SZV-1287** |  |  |  |  |  |
| *p* value | 0.6402 | 0.4996 | 0.8990 | 0.6124 | 0.7367 |
| Hedges’ *g* (effect size) | **0.6 ↓** | **0.7 ↓** | 0.3 | **0.6 ↓** | 0.5 |

**Supplementary table 13. Anteroposterior diameters of the ipsilateral knees of vehicle-treated mice.** The table shows the means of anteroposterior diameters of the ipsilateral knees of vehicle-treated saline-, 0.5 mg and 0.8 mg monoiodoacetate (MIA)-injected mice with 95% CI of n=7-29 mice/group.

| Time | **Saline** | | | **0.5 mg MIA** | | | **0.8 mg MIA** | | |
| --- | --- | --- | --- | --- | --- | --- | --- | --- | --- |
|  | Mean (mm) | Upper limit | Lower limit | Mean (mm) | Upper limit | Lower limit | Mean (mm) | Upper limit | Lower limit |
|  |  |  |  |  |  |  |  |  |  |
| **Baseline** | 4.73 | 4.98 | 4.47 | 4.65 | 4.74 | 4.55 | 4.92 | 5.02 | 4.82 |
|  |  |  |  |  |  |  |  |  |  |
| **Hour 3** | 4.91 | 5.31 | 4.51 | 5.45 | 5.59 | 5.31 | 5.87 | 6.32 | 5.43 |
|  |  |  |  |  |  |  |  |  |  |
| **Hour 6** | 4.85 | 5.29 | 4.41 | 5.85 | 6.03 | 5.66 | 6.16 | 6.62 | 5.70 |
|  |  |  |  |  |  |  |  |  |  |
| **Day 1** | 5.00 | 5.36 | 4.65 | 5.83 | 5.98 | 5.67 | 5.92 | 6.54 | 5.30 |
|  |  |  |  |  |  |  |  |  |  |
| **Day 2** | 4.84 | 5.08 | 4.60 | 5.32 | 5.47 | 5.17 | 5.72 | 6.15 | 5.29 |
|  |  |  |  |  |  |  |  |  |  |
| **Day 9** | 4.84 | 5.23 | 4.45 | 4.94 | 5.14 | 4.73 | 5.34 | 5.51 | 5.17 |

**Supplementary table 14. Mediolateral diameters of the ipsilateral knees of vehicle-treated mice.** The table shows the means of mediolateral diameters of the ipsilateral knees of vehicle-treated saline-, 0.5 mg and 0.8 mg monoiodoacetate (MIA)-injected mice with 95% CI of n=7-29 mice/group.

| Time | **Saline** | | | **0.5 mg MIA** | | | **0.8 mg MIA** | | |
| --- | --- | --- | --- | --- | --- | --- | --- | --- | --- |
|  | Mean (mm) | Upper limit | Lower limit | Mean (mm) | Upper limit | Lower limit | Mean (mm) | Upper limit | Lower limit |
|  |  |  |  |  |  |  |  |  |  |
| **Baseline** | 3.84 | 3.98 | 3.69 | 4.03 | 4.16 | 3.90 | 4.47 | 4.60 | 4.34 |
|  |  |  |  |  |  |  |  |  |  |
| **Hour 3** | 3.75 | 4.02 | 3.48 | 4.88 | 5.10 | 4.66 | 5.66 | 5.89 | 5.42 |
|  |  |  |  |  |  |  |  |  |  |
| **Hour 6** | 4.00 | 4.45 | 3.54 | 5.25 | 5.49 | 5.01 | 5.90 | 6.20 | 5.59 |
|  |  |  |  |  |  |  |  |  |  |
| **Day 1** | 4.05 | 4.47 | 3.63 | 5.14 | 5.39 | 4.89 | 5.66 | 5.99 | 5.32 |
|  |  |  |  |  |  |  |  |  |  |
| **Day 2** | 3.76 | 3.92 | 3.59 | 4.60 | 4.75 | 4.45 | 5.58 | 5.81 | 5.36 |
|  |  |  |  |  |  |  |  |  |  |
| **Day 9** | 3.90 | 4.15 | 3.64 | 4.19 | 4.34 | 4.05 | 5.00 | 5.29 | 4.70 |

**Supplementary table 15 Anteroposterior diameters of the ipsilateral knees of SZV-1287-treated mice.** The table shows the means of anteroposterior diameters of the ipsilateral knees of SZV-1287-treated (20 mg/kg i.p. every day during the 21-day experimental period) 0.5 mg and 0.8 mg monoiodoacetate (MIA)-injected mice with 95% CI of n=7-29 mice/group.

| Time | **Saline** | | | **0.5 mg MIA** | | | **0.8 mg MIA** | | |
| --- | --- | --- | --- | --- | --- | --- | --- | --- | --- |
|  | Mean (mm) | Upper limit | Lower limit | Mean (mm) | Upper limit | Lower limit | Mean (mm) | Upper limit | Lower limit |
|  |  |  |  |  |  |  |  |  |  |
| **Baseline** | 4.70 | 4.89 | 4.52 | 4.69 | 4.79 | 4.59 | 4.80 | 5.01 | 4.60 |
|  |  |  |  |  |  |  |  |  |  |
| **Hour 3** | 4.69 | 4.85 | 4.52 | 5.54 | 5.69 | 5.38 | 5.64 | 6.18 | 5.11 |
|  |  |  |  |  |  |  |  |  |  |
| **Hour 6** | 4.72 | 5.03 | 4.42 | 6.02 | 6.22 | 5.81 | 5.82 | 6.21 | 5.42 |
|  |  |  |  |  |  |  |  |  |  |
| **Day 1** | 4.78 | 4.92 | 4.63 | 5.81 | 6.02 | 5.60 | 5.62 | 6.00 | 5.25 |
|  |  |  |  |  |  |  |  |  |  |
| **Day 2** | 4.78 | 4.90 | 4.67 | 5.29 | 5.45 | 5.13 | 5.76 | 5.85 | 5.68 |
|  |  |  |  |  |  |  |  |  |  |
| **Day 9** | 4.64 | 4.81 | 4.48 | 4.96 | 5.20 | 4.72 | 5.16 | 5.44 | 4.88 |

**Supplementary table 16. Mediolateral diameters of the ipsilateral knees of SZV-1287-treated mice.** The table shows the means of mediolateral diameters of the ipsilateral knees of SZV-1287-treated (20 mg/kg i.p. every day during the 21-day experimental period) 0.5 mg and 0.8 mg monoiodoacetate (MIA)-injected mice with 95% CI of n=7-29 mice/group.

| Time | **Saline** | | | **0.5 mg MIA** | | | **0.8 mg MIA** | | |
| --- | --- | --- | --- | --- | --- | --- | --- | --- | --- |
|  | Mean (mm) | Upper limit | Lower limit | Mean (mm) | Upper limit | Lower limit | Mean (mm) | Upper limit | Lower limit |
|  |  |  |  |  |  |  |  |  |  |
| **Baseline** | 3.72 | 3.95 | 3.49 | 3.99 | 4.12 | 3.85 | 4.48 | 4.59 | 4.37 |
|  |  |  |  |  |  |  |  |  |  |
| **Hour 3** | 3.69 | 3.87 | 3.50 | 4.94 | 5.17 | 4.71 | 5.41 | 5.74 | 5.08 |
|  |  |  |  |  |  |  |  |  |  |
| **Hour 6** | 3.81 | 3.98 | 3.64 | 5.43 | 5.70 | 5.17 | 5.58 | 5.92 | 5.25 |
|  |  |  |  |  |  |  |  |  |  |
| **Day 1** | 3.81 | 3.97 | 3.65 | 5.14 | 5.34 | 4.94 | 5.52 | 5.79 | 5.24 |
|  |  |  |  |  |  |  |  |  |  |
| **Day 2** | 3.76 | 3.92 | 3.60 | 4.63 | 4.80 | 4.45 | 5.39 | 5.60 | 5.19 |
|  |  |  |  |  |  |  |  |  |  |
| **Day 9** | 3.73 | 3.87 | 3.58 | 4.20 | 4.39 | 4.00 | 4.84 | 5.06 | 4.63 |

**Supplementary table 17. Comparison of the composite arthritis scores of the knee joints.** The table shows the statistics for composite arthritis scores of the contra- and ipsilateral knee joints of vehicle- and SZV-1287-treated (20 mg/kg i.p. every day during the 21-day experimental period) saline-, 0.5 mg and 0.8 mg monoiodoacetate (MIA)-injected mice on day 22. The statistics were performed using box plots of composite score for n=6-15 mice/group. *P*<0.05 (Kruskal-Wallis test followed by Dunn’s multiple comparisons test) was considered significant indicated by * and effect size was calculated using Hedges’ *g*.

| **Group comparisons** | **Contralateral** | **Ipsilateral** |
| --- | --- | --- |
|  |  |  |
| **Saline vs. 0.5 mg MIA Vehicle** |  |  |
| *p* value | >0.9999 | **0.0275*** |
| Hedges’ *g* (effect size) | 0.3 | **1.9 ↑** |
|  |  |  |
| **Saline vs. 0.8 mg MIA Vehicle** |  |  |
| *p* value | 0.0735 | **0.0132*** |
| Hedges’ *g* (effect size) | **1.9 ↑** | **1.9 ↑** |
|  |  |  |
| **0.5 mg vs. 0.8 mg MIA Vehicle** |  |  |
| *p* value | 0.1772 | >0.9999 |
| Hedges’ *g* (effect size) | **0.9 ↑** | 0.2 |
|  |  |  |
| **Saline vs. 0.5 mg MIA SZV-1287** |  |  |
| *p* value | >0.9999 | >0.9999 |
| Hedges’ *g* (effect size) | 0 | **0.7 ↑** |
|  |  |  |
| **Saline vs. 0.8 mg MIA SZV-1287** |  |  |
| *p* value | 0.1672 | 0.0524 |
| Hedges’ *g* (effect size) | **2.0 ↑** | **3.3 ↑** |
|  |  |  |
| **0.5 mg vs. 0.8 mg MIA SZV-1287** |  |  |
| *p* value | 0.0682 | 0.3407 |
| Hedges’ *g* (effect size) | **1.3 ↑** | **1 ↑** |
|  |  |  |
| **0.5 mg MIA Vehicle vs. SZV-1287** |  |  |
| *p* value | >0.9999 | 0.0506 |
| Hedges’ *g* (effect size) | 0 | **1.1 ↓** |
|  |  |  |
| **0.8 mg MIA Vehicle SZV-1287** |  |  |
| *p* value | >0.9999 | >0.9999 |
| Hedges’ *g* (effect size) | 0.3 | 0.4 |

**Supplementary table 18. Comparison of the composite arthritis scores of the ipsilateral knee joints with the contralateral side.** The table shows the statistics for composite arthritis scores of the ipsilateral knee joints of vehicle- and SZV-1287-treated (20 mg/kg i.p. every day during the 21-day experimental period) saline-, 0.5 mg and 0.8 mg monoiodoacetate (MIA)-injected mice as compared to the respective contralateral side on day 22. The statistics were performed using box plots of composite score for n=6-15 mice/group. *P*<0.05 (Kruskal-Wallis test followed by Dunn’s multiple comparisons test) was considered significant indicated by * and effect size was calculated using Hedges’ *g*.

| **Groups** | **In comparison with the contralateral side** |
| --- | --- |
|  |  |
| **Saline Vehicle** |  |
| *p* value | >0.9999 |
| Hedges’ *g* (effect size) | 0 |
|  |  |
| **0.5 mg MIA Vehicle** |  |
| *p* value | **0.0469*** |
| Hedges’ *g* (effect size) | **1.3 ↑** |
|  |  |
| **0.8 mg MIA Vehicle** |  |
| *p* value | >0.9999 |
| Hedges’ *g* (effect size) | **0.8 ↑** |
|  |  |
| **Saline SZV-1287** |  |
| *p* value | >0.9999 |
| Hedges’ *g* (effect size) | 0.2 |
|  |  |
| **0.5 mg MIA SZV-1287** |  |
| *p* value | >0.9999 |
| Hedges’ *g* (effect size) | **0.6 ↑** |
|  |  |
| **0.8 mg MIA SZV-1287** |  |
| *p* value | >0.9999 |
| Hedges’ *g* (effect size) | **0.5 ↑** |

**Supplementary table 19. Comparison of the neutrophil myeloperoxidase (MPO) activity, matrix metalloproteinase (MMP) activity, vascular leakage, and bone remodeling of the ipsilateral knees.** The table shows the statistics for neutrophil MPO activity, MMP activity, inflammatory vascular changes, and bone remodeling of the ipsilateral knee joints of vehicle- and SZV-1287-treated (20 mg/kg i.p. every day during the 21-day experimental period) 0.5 mg monoiodoacetate (MIA)-injected mice as compared to the contralateral side. The statistics were performed using the means with 95% CI of n=6-12 mice in the case of neutrophil MPO activity, n=5-6 mice in the case of MMP activity, n=5-6 in the case of inflammatory vascular changes and n=3-8 in the case of bone remodeling. *P*<0.05 (2-way ANOVA followed by Sidak’s multiple comparisons test) was considered significant indicated by * and effect size was calculated using Hedges’ *g*.

| **Variable** | **In comparison with the contralateral side** | |
| --- | --- | --- |
|  | ***p* value** | **Hedges’ *g***  **(effect size)** |
|  |  |  |
| **Neutrophil MPO activity** |  |  |
| Hour 3 Vehicle | **0.0014**** | **1.9 ↑** |
| Hour 3 SZV-1287 | 0.0918 | **1.7 ↑** |
| Hour 24 Vehicle | **<0.0001****** | **3.1 ↑** |
| Hour 24 SZV-1287 | **0.0012***** | **2 ↑** |
|  |  |  |
| **MMP activity** |  |  |
| Day 4 Vehicle | **0.0001***** | **1.2 ↑** |
| Day 4 SZV-1287 | **<0.0001****** | **0.6 ↑** |
| Day 18 Vehicle | **0.0185*** | 0.4 |
| Day 18 SZV-1287 | 0.1788 | 0.1 |
|  |  |  |
| **Vascular leakage** (Day 7) |  |  |
| Vehicle | **<0.0001****** | 0.3 |
| SZV-1287 | **0.0002***** | 0.5 |
| **Bone remodeling** (Day 22) |  |  |
| Vehicle | **0.0145*** | 0.4 |
| SZV-1287 | **0.0056**** | **1.2 ↑** |

**Supplementary table 20. Comparison of the neutrophil myeloperoxidase (MPO) activity, matrix metalloproteinase (MMP) activity, vascular leakage, and bone remodeling of the ipsilateral knees of vehicle- and SZV-1287-treated osteoarthritic mice.** The table shows the statistics for neutrophil MPO activity, MMP activity, inflammatory vascular changes, and bone remodeling of the ipsilateral knee joints SZV-1287-treated (20 mg/kg i.p. every day during the 21-day experimental period) 0.5 mg monoiodoacetate (MIA)-injected mice as compared to the vehicle-treated group. The statistics were performed using the means with 95% CI of n=6-12 mice in the case of neutrophil MPO activity, n=5-6 mice in the case of MMP activity, n=5-6 in the case of inflammatory vascular changes and n=3-8 in the case of bone remodeling. *P*<0.05 (2-way ANOVA followed by Sidak’s multiple comparisons test) was considered significant indicated by * and effect size was calculated using Hedges’ *g*.

| **Variable** | **In comparison with the vehicle-treated group** | |
| --- | --- | --- |
|  | ***p* value** | **Hedges’ *g***  **(effect size)** |
|  |  |  |
| **Neutrophil MPO activity** |  |  |
| Hour 3 | **0.0247*** | **1.2 ↓** |
| Hour 24 | **<0.0001****** | **1.7 ↓** |
|  |  |  |
| **MMP activity** |  |  |
| Day 4 | 0.9920 | 0 |
| Day 18 | 0.7860 | 0.1 |
|  |  |  |
| **Vascular leakage** (Day 7) | 0.7727 | 0 |
|  |  |  |
| **Bone remodeling** (Day 22) | 0.7062 | 0.4 |

**Supplementary table 21. Comparison of the bone morphology of the distal femur and proximal tibia of vehicle- and SZV-1287-treated osteoarthritic mice.** The table shows the statistics of microarchitectural parameters of contra- and ipsilateral distal femora and proximal tibiae of SZV-1287-treated (20 mg/kg i.p. every day during the 21-day experimental period) 0.5 mg MIA-injected mice as compared to the vehicle-treated group on day 22. The statistics were performed using the means with 95% CI of n=8-10 mice/group. *P*<0.05 (two-way ANOVA followed by Sidak’s multiple comparisons test) was considered significant and effect size was calculated using Hedges’ *g*. BV/TV, bone volume/total volume (bone volume density); Tb.Sp, trabecular separation; Tb.N, trabecular number; Tb.Pf, trabecular pattern factor; Po.V(op), volume of open pore space; Po(op), open porosity.

| **Variable** | **In comparison with the vehicle-treated mice** | | | |
| --- | --- | --- | --- | --- |
|  | **Distal femur** | | **Proximal tibia** | |
|  | **Contralateral** | **Ipsilateral** | **Contralateral** | **Ipsilateral** |
|  |  |  |  |  |
| **BV/TV** |  |  |  |  |
| *p* value | 0.0513 | 0.4674 | 0.2010 | 0.2815 |
| Hedges’ *g* (effect size) | **1.3 ↓** | 0.5 | **0.8 ↓** | **0.7 ↓** |
|  |  |  |  |  |
| **Tb.N** |  |  |  |  |
| *p* value | >0.9999 | 0.9441 | 0.6351 | >0.9999 |
| Hedges’ *g* (effect size) | 0 | 0.2 | 0.3 | 0 |
|  |  |  |  |  |
| **Tb.Sp** |  |  |  |  |
| *p* value | 0.9292 | 0.7955 | 0.4008 | 0.8338 |
| Hedges’ *g* (effect size) | 0.2 | 0.3 | **0.6 ↓** | 0.3 |
|  |  |  |  |  |
| **Tb.Pf** |  |  |  |  |
| *p* value | 0.6186 | 0.6946 | 0.6818 | 0.5797 |
| Hedges’ *g* (effect size) | 0.3 | 0.5 | 0.3 | 0.5 |
|  |  |  |  |  |
| **Po.V(op)** |  |  |  |  |
| *p* value | 0.2249 | 0.9255 | 0.9679 | 0.7947 |
| Hedges’ *g* (effect size) | **1 ↑** | 0.1 | 0.1 | 0.3 |
|  |  |  |  |  |
| **Po(op)** |  |  |  |  |
| *p* value | 0.0501 | 0.4715 | 0.1928 | 0.3220 |
| Hedges’ *g* (effect size) | **1.3 ↑** | 0.1 | **0.8 ↑** | **0.7 ↑** |

**Supplementary table 22. Comparison of the bone morphology of the distal femur and proximal tibia of vehicle- and SZV-1287-treated osteoarthritic mice.** The table shows the statistics of microarchitectural parameters of ipsilateral distal femora and proximal tibiae of vehicle- and SZV-1287-treated (20 mg/kg i.p. every day during the 21-day experimental period) 0.5 mg MIA-injected mice as compared to the respective contralateral side on day 22. The statistics were performed using the means with 95% CI of n=8-10 mice/group. *P*<0.05 (two-way ANOVA followed by Sidak’s multiple comparison test) was considered significant and effect size was calculated using Hedges’ *g*. BV/TV, bone volume/total volume (bone volume density); Tb.Sp, trabecular separation; Tb.N, trabecular number; Tb.Pf, trabecular pattern factor; Po.V(op), volume of open pore space; Po(op), open porosity.

| **Variable** | **In comparison with the contralateral side** | | | |
| --- | --- | --- | --- | --- |
|  | **Distal femur** | | **Proximal tibia** | |
|  | **Vehicle** | **SZV-1287** | **Vehicle** | **SZV-1287** |
|  |  |  |  |  |
| **BV/TV** |  |  |  |  |
| *p* value | **0.0066**** | 0.1924 | 0.2606 | 0.4404 |
| Hedges’ *g* (effect size) | **1.5** **↓** | **0.8 ↓** | **0.7 ↓** | 0.5 |
|  |  |  |  |  |
| **Tb.N** |  |  |  |  |
| *p* value | **0.0477*** | 0.1509 | 0.2790 | 0.0820 |
| Hedges’ *g* (effect size) | **1 ↓** | **0.9 ↓** | **0.6** **↓** | **1.1 ↓** |
|  |  |  |  |  |
| **Tb.Sp** |  |  |  |  |
| *p* value | 0.1060 | 0.6196 | 0.7580 | 0.3883 |
| Hedges’ *g* (effect size) | **0.9** **↑** | 0.4 | 0.3 | **0.8 ↑** |
|  |  |  |  |  |
| **Tb.Pf** |  |  |  |  |
| *p* value | **<0.0001****** | **0.0002***** | 0.1259 | 0.1398 |
| Hedges’ *g* (effect size) | **2.5** **↑** | **2 ↑** | **1.2** **↑** | **0.7 ↑** |
|  |  |  |  |  |
| **Po.V(op)** |  |  |  |  |
| *p* value | **0.0178*** | 0.3613 | 0.5458 | 0.3904 |
| Hedges’ *g* (effect size) | **1.2 ↑** | **0.7 ↑** | 0.4 | **0.7 ↑** |
|  |  |  |  |  |
| **Po(op)** |  |  |  |  |
| *p* value | **0.0065**** | 0.1951 | 0.2431 | 0.4799 |
| Hedges’ *g* (effect size) | **1.4** **↑** | **0.8 ↑** | **0.7 ↑** | 0.5 |

**Supplementary table 23. Comparison of the astrocyte and microglia densities of the contra- and ipsilateral lumbar dorsal horn of the spinal cord with the saline-injected group.** The table shows the statistics of astrocyte and microglia density in the contra- and ipsilateral L4-L6 spinal dorsal horn of vehicle- and SZV-1287-treated (20 mg/kg i.p. every day during the 21-day experimental period) 0.5 mg MIA-injected mice as compared to the saline-injected group on day 22. The statistics were performed using the means with 95% CI of n=5-8 mice/group, 2-4 sections/mouse. *P*<0.05 (two-way ANOVA followed by Sidak’s multiple comparisons test) was considered significant indicated by * and effect size was calculated using Hedges’ *g*.

| **Variable** | **In comparison with the saline-injected group** | |
| --- | --- | --- |
|  | **Contralateral** | **Ipsilateral** |
|  |  |  |
| **GFAP density Vehicle** |  |  |
| *p* value | **0.0088**** | 0.0648 |
| Hedges’ *g* (effect size) | **1.7** **↑** | **1.4 ↑** |
|  |  |  |
| **Iba1 density Vehicle** |  |  |
| *p* value | 0.8232 | 0.3528 |
| Hedges’ *g* (effect size) | 0.4 | **0.8 ↑** |
|  |  |  |
| **GFAP density SZV-1287** |  |  |
| *p* value | 0.8779 | 0.9941 |
| Hedges’ *g* (effect size) | 0.2 | 0.1 |
|  |  |  |
| **Iba1 density SZV-1287** |  |  |
| *p* value | **0.0303*** | 0.0909 |
| Hedges’ *g* (effect size) | **1.7 ↑** | **1 ↑** |

**Supplementary table 24. Comparison of the astrocyte and microglia densities of the ipsilateral lumbar dorsal horn of the spinal cord with the contralateral side.** The table shows the statistics of astrocyte and microglia density in the ipsilateral L4-L6 spinal dorsal horn of vehicle- and SZV-1287-treated (20 mg/kg i.p. every day during the 21-day experimental period) saline- and 0.5 mg MIA-injected mice as compared to the respective contralateral side on day 22. The statistics were performed using the means with 95% CI of n=5-8 mice/group, 2-4 sections/mouse. *P*<0.05 (two-way ANOVA followed by Sidak’s multiple comparisons test) was considered significant indicated by * and effect size was calculated using Hedges’ *g*.

| **Variable** | **In comparison with the contralateral side** | |
| --- | --- | --- |
|  | **Saline** | **MIA** |
|  |  |  |
| **GFAP density Vehicle** |  |  |
| *p* value | 0.7553 | 0.9835 |
| Hedges’ *g* (effect size) | **0.7 ↑** | 0.1 |
|  |  |  |
| **Iba1 density Vehicle** |  |  |
| *p* value | 0.9834 | 0.7805 |
| Hedges’ *g* (effect size) | 0.1 | 0.3 |
|  |  |  |
| **GFAP density SZV-1287** |  |  |
| *p* value | 0.9809 | 0.9788 |
| Hedges’ *g* (effect size) | 0.1 | 0.1 |
|  |  |  |
| **Iba1 density SZV-1287** |  |  |
| *p* value | 0.9006 | 0.9904 |
| Hedges’ *g* (effect size) | 0.2 | 0.1 |

**Supplementary table 25. Comparison of the astrocyte and microglia densities of the contra- and ipsilateral lumbar dorsal horn of the spinal cord of vehicle- and SZV-1287-treated osteoarthritic mice.** The table shows the statistics of astrocyte and microglia density in the contra- and ipsilateral L4-L6 spinal dorsal horn of SZV-1287-treated (20 mg/kg i.p. every day during the 21-day experimental period) 0.5 mg MIA-injected mice as compared to the vehicle-treated group on day 22. The statistics were performed using the means with 95% CI of n=5-8 mice/group, 2-4 sections/mouse. *P*<0.05 (two-way ANOVA followed by Sidak’s multiple comparison test) was considered significant indicated by * and effect size was calculated using Hedges’ *g*.

| **Variable** | **In comparison with the vehicle-treated group** | |
| --- | --- | --- |
|  | **Contralateral** | **Ipsilateral** |
|  |  |  |
| **GFAP density** |  |  |
| *p* value | 0.1913 | 0.1015 |
| Hedges’ *g* (effect size) | **0.8 ↓** | **1 ↓** |
|  |  |  |
| **Iba1 density** |  |  |
| *p* value | 0.3307 | 0.0863 |
| Hedges’ *g* (effect size) | **0.8 ↓** | **1.3 ↓** |

**Supplementary table 26. Comparison of the astrocyte and microglia densities of the periaqueductal grey with the saline-injected group.** The table shows the statistics of astrocyte and microglia density in the periaqueductal grey of vehicle-treated 0.5 mg MIA-injected mice as compared to the saline-injected group on day 22. The statistics were performed using the means with 95% CI of n=5-11 mice/group, 2-4 sections/mouse. *P*<0.05 (unpaired t test) was considered significant indicated by * and effect size was calculated using Hedges’ *g*.

| **Variable** | **In comparison with the saline-injected group** |
| --- | --- |
|  |  |
| **GFAP density** |  |
| *p* value | 0.8119 |
| Hedges’ *g* (effect size) | 0.1 |
|  |  |
| **Iba1 density** |  |
| *p* value | 0.5819 |
| Hedges’ *g* (effect size) | 0.3 |

**Supplementary table 27. Comparison of the astrocyte and microglia densities in the somatosensory cortex with the saline-injected group.** The table shows the statistics of astrocyte density in the contra- and ipsilateral somatosensory cortex of vehicle-treated 0.5 mg MIA-injected mice as compared to the saline-injected group on day 22. The statistics were performed using the means with 95% CI of n=5-8 mice/group, 2-4 sections/mouse. *P*<0.05 (two-way ANOVA followed by Sidak’s multiple comparisons test) was considered significant indicated by * and effect size was calculated using Hedges’ *g*.

| **Variable** | **In comparison with the saline-injected group** | |
| --- | --- | --- |
|  | **Contralateral** | **Ipsilateral** |
|  |  |  |
| **GFAP density** |  |  |
| *p* value | 0.8603 | 0.9472 |
| Hedges’ *g* (effect size) | 0.3 | 0.2 |
|  |  |  |
| **Iba1 density** |  |  |
| *p* value | 0.9987 | 0.6671 |
| Hedges’ *g* (effect size) | 0 | 0.5 |

**Supplementary table 28. Comparison of the astrocyte and microglia densities of the somatosensory cortex with the contralateral side.** The table shows the statistics of astrocyte density in the ipsilateral L4-L6 spinal dorsal horn of vehicle-treated saline- and 0.5 mg MIA-injected mice as compared to the respective contralateral side on day 22. The statistics were performed using the means with 95% CI of n=5-8 mice/group, 2-4 sections/mouse. *P*<0.05 (two-way ANOVA followed by Sidak’s multiple comparisons test) was considered significant indicated by * and effect size was calculated using Hedges’s *g*.

| **Variable** | **In comparison with the contralateral side** | |
| --- | --- | --- |
|  | **Saline** | **MIA** |
|  |  |  |
| **GFAP density** |  |  |
| *p* value | 0.7316 | 0.5548 |
| Hedges’ *g* (effect size) | 0.5 | 0.5 |
|  |  |  |
| **Iba1 density** |  |  |
| *p* value | 0.5871 | 0.9826 |
| Hedges’ *g* (effect size) | 0.5 | 0.1 |
