# Supplementary figures and images for: Novel multitarget analgesic candidate SZV-1287 demonstrates potential disease-modifying effects in the monoiodoacetate-induced osteoarthritis mouse model
Source: Front Pharmacol. 2024 Sep 16;15:1377081. doi: 10.3389/fphar.2024.1377081 (PMC11439770; doi:10.3389/fphar.2024.1377081)

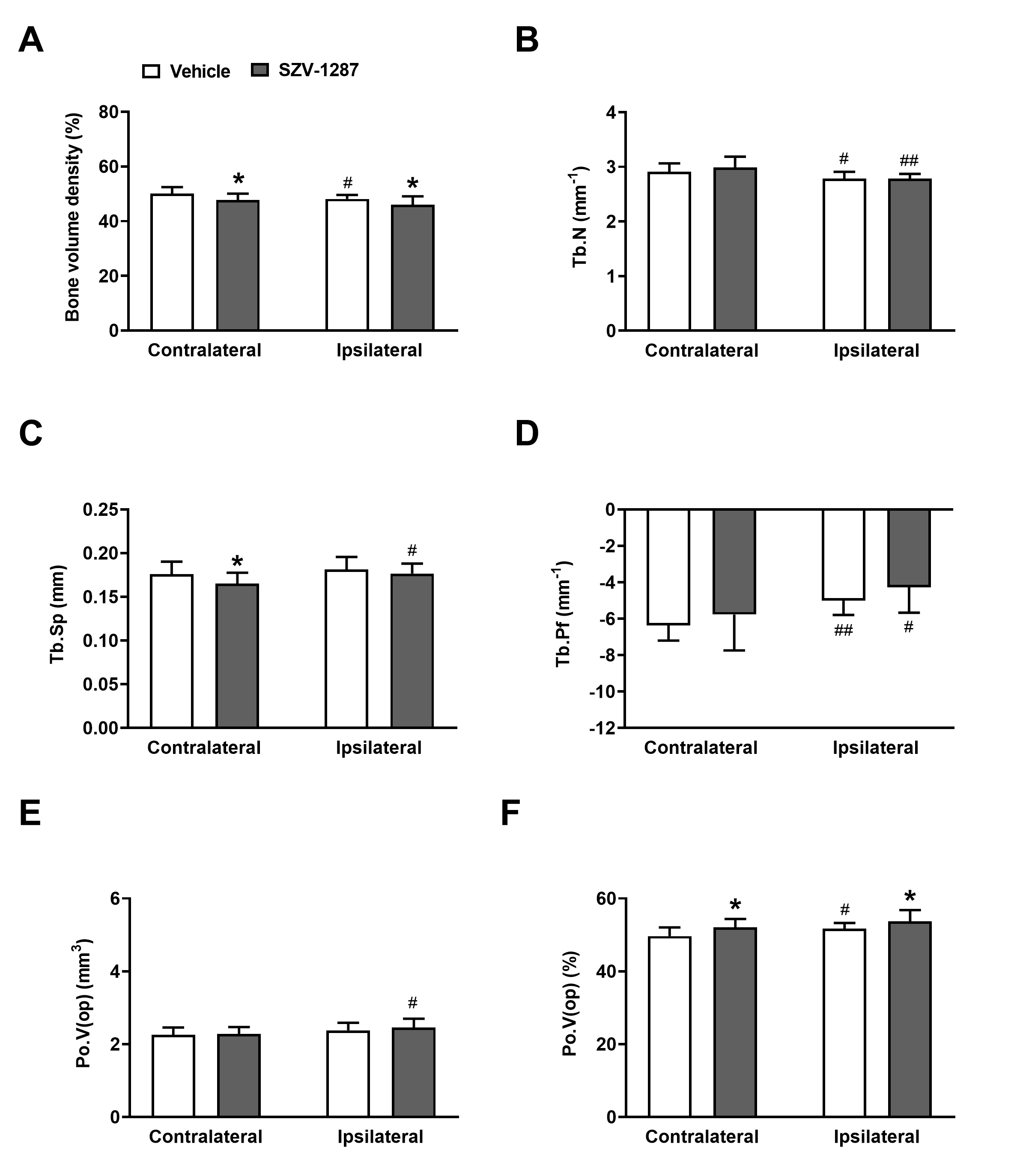

Supplement: Supplementary file 2 [file Image3.TIF]

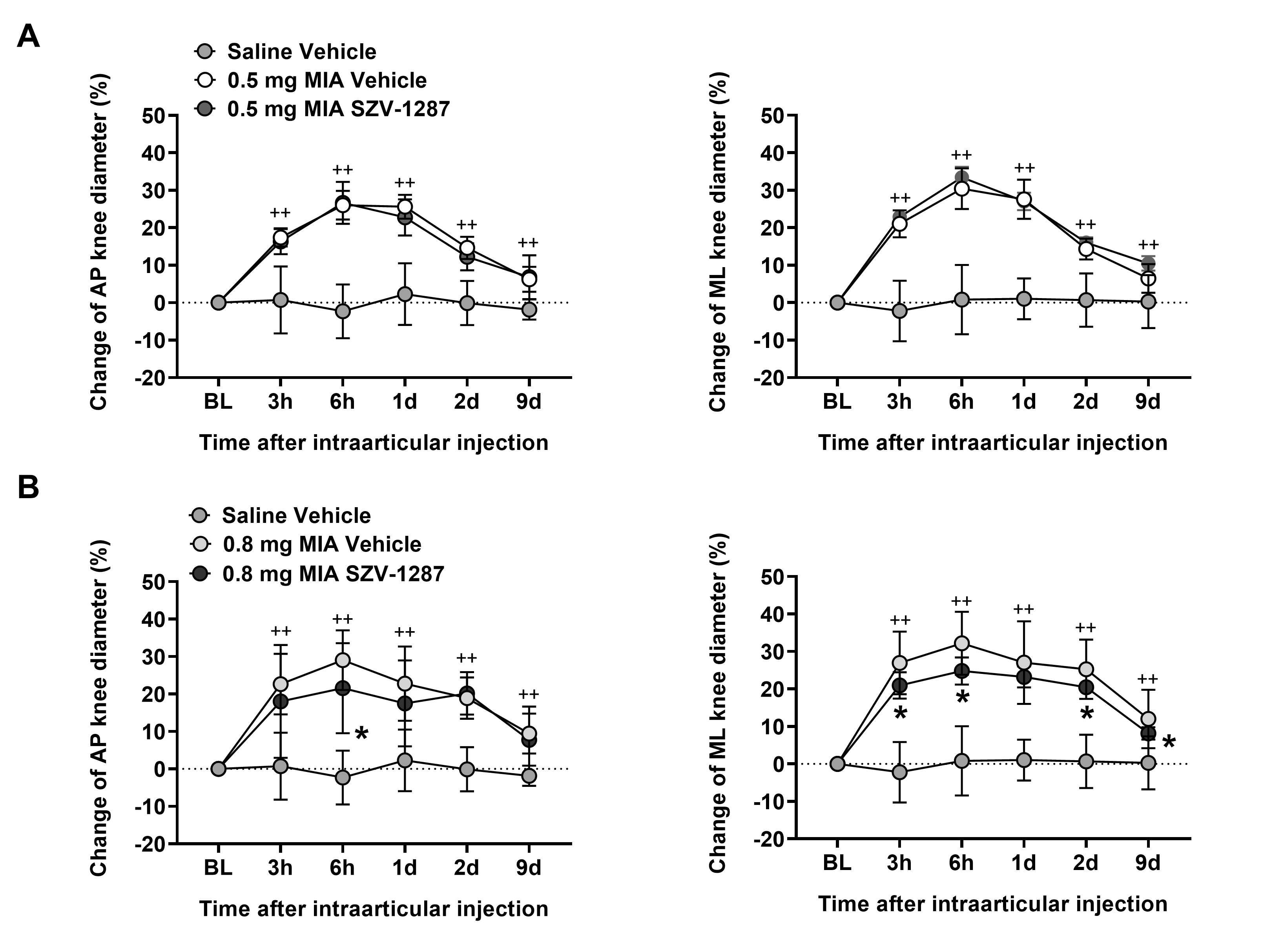

Supplement: Supplementary file 3 [file Image2.TIF]

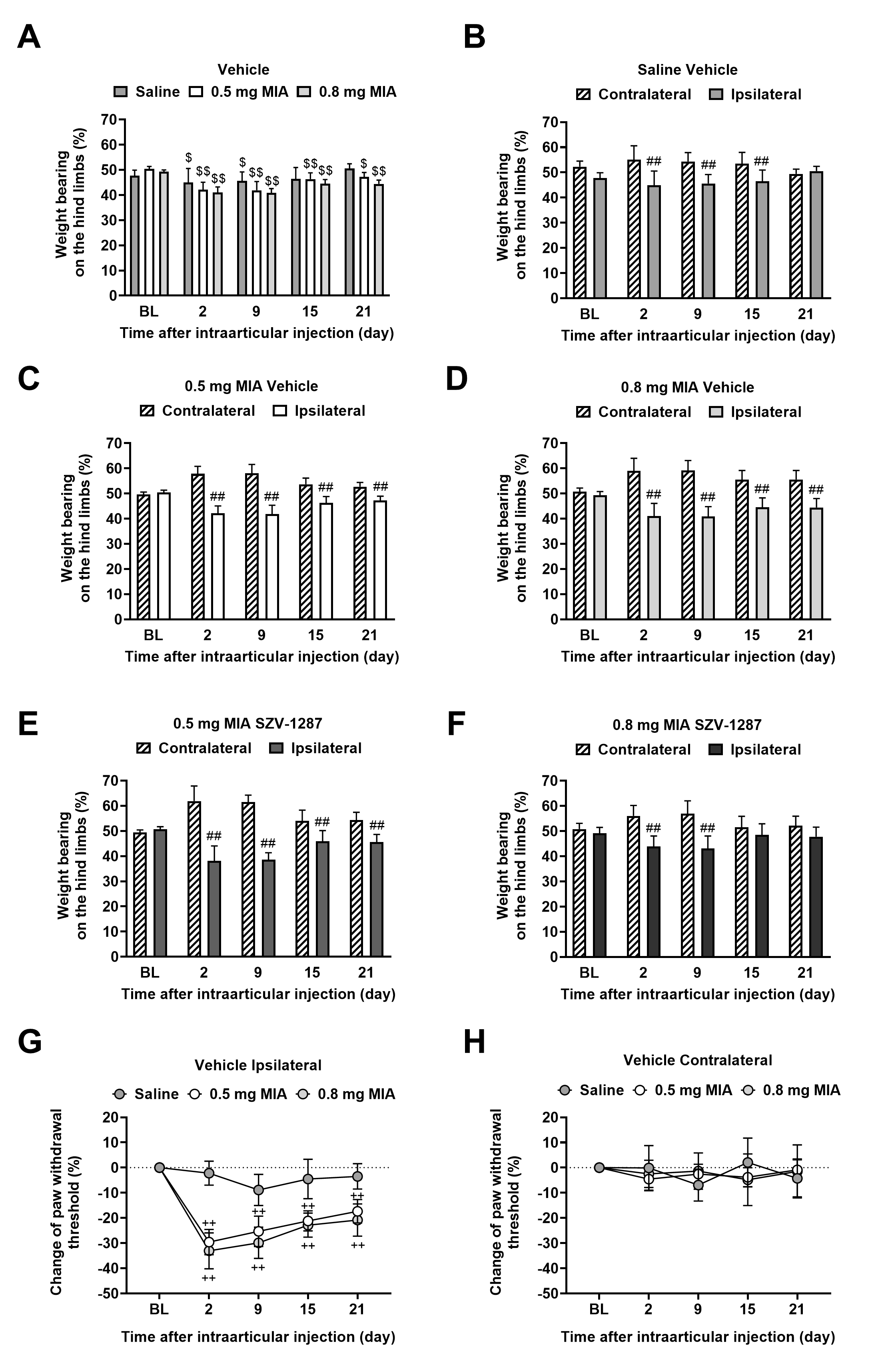

Supplement: Supplementary file 4 [file Image1.TIF]
